# Disclosing the molecular profile of the human amniotic mesenchymal stromal cell secretome by filter-aided sample preparation proteomic characterization

**DOI:** 10.1186/s13287-023-03557-4

**Published:** 2023-11-27

**Authors:** Alexandra Muntiu, Andrea Papait, Federica Vincenzoni, Alberto Vitali, Wanda Lattanzi, Pietro Romele, Anna Cargnoni, Antonietta Silini, Ornella Parolini, Claudia Desiderio

**Affiliations:** 1 Istituto di Scienze e Tecnologie Chimiche (SCITEC) ‘’Giulio Natta’’, Consiglio Nazionale delle Ricerche, Rome, Italy; 2 Department of Life Science and Public Health, Università Cattolica del Sacro Cuore, Rome, Italy; 3 Fondazione Policlinico Universitario ‘’Agostino Gemelli’’ Istituto di Ricovero e Cura a Carattere Scientifico, IRCCS, Rome, Italy; 4 Dipartimento di Scienze Biotecnologiche di Base, Cliniche Intensivologiche e Perioperatorie, Università Cattolica del Sacro Cuore, Rome, Italy; 5 Centro di Ricerca E. Menni, Fondazione Poliambulanza Istituto Ospedaliero, Brescia, Italy

**Keywords:** Proteomics, Secretome, Human amniotic mesenchymal stromal cells, Filter-aided sample preparation, Immunomodulation, Regenerative medicine

## Abstract

**Background:**

The secretome of mesenchymal stromal cells isolated from the amniotic membrane (hAMSCs) has been extensively studied for its in vitro immunomodulatory activity as well as for the treatment of several preclinical models of immune-related disorders. The bioactive molecules within the hAMSCs secretome are capable of modulating the immune response and thus contribute to stimulating regenerative processes. At present, only a few studies have attempted to define the composition of the secretome, and several approaches, including multi-omics, are underway in an attempt to precisely define its composition and possibly identify key factors responsible for the therapeutic effect.

**Methods:**

In this study, we characterized the protein composition of the hAMSCs secretome by a filter-aided sample preparation (FASP) digestion and liquid chromatography-high resolution mass spectrometry (LC–MS) approach. Data were processed for gene ontology classification and functional protein interaction analysis by bioinformatics tools.

**Results:**

Proteomic analysis of the hAMSCs secretome resulted in the identification of 1521 total proteins, including 662 unique elements. A number of 157 elements, corresponding to 23.7%, were found as repeatedly characterizing the hAMSCs secretome, and those that resulted as significantly over-represented were involved in immunomodulation, hemostasis, development and remodeling of the extracellular matrix molecular pathways.

**Conclusions:**

Overall, our characterization enriches the landscape of hAMSCs with new information that could enable a better understanding of the mechanisms of action underlying the therapeutic efficacy of the hAMSCs secretome while also providing a basis for its therapeutic translation.

**Supplementary Information:**

The online version contains supplementary material available at 10.1186/s13287-023-03557-4.

## Introduction

Mesenchymal stromal cells isolated from the amniotic membrane (hAMSCs) of human term placenta possess potent immunomodulatory activity. Unlike other MSC, these cells do not require priming to exert their action on the cells of the immune system [1]. Furthermore, hAMSCs are readily available from biological waste at the time of delivery without posing any risk to the donor, and a large number of cells can be obtained from human term placenta [2].

It has been widely demonstrated that hAMSCs modulate the immune response mediated by both innate and adaptive immune cells. Indeed, hAMSCs inhibit the proliferation and differentiation of T lymphocytes toward inflammatory and cytotoxic subsets while promoting polarization toward regulatory T cells [3–5]. Furthermore, hAMSCs also modulate the polarization of monocytes to antigen presenting cells (mature dendritic cells and inflammatory macrophages) by inducing the acquisition of features that are typical of M2 immunoregulatory macrophages [4–6]. In addition, hAMSCs suppress the proliferation and differentiation of B lymphocytes to plasma cells [7]. The in vitro immunomodulatory properties exhibited by hAMSCs are reflected in their therapeutic efficacy in various disease models characterized by impaired immune responses. In both in vitro and in vivo studies, the administration of conditioned medium collected from hAMSCs demonstrated comparable therapeutic efficacy to that of hAMSCs themselves [1, 4, 8–12]. These findings emphasize how the therapeutic actions of hAMSCs are primarily determined by their paracrine activity.

The mechanisms through which hAMSCs regulate the immune response and enable other cells to facilitate tissue repair during pathological processes are only partially understood. The factors produced by hAMSCs that orchestrate their beneficial properties currently remain an intriguing subject of investigation. To date, the characterization of the hAMSCs secretome is limited to a few studies that employ various methodological approaches. For example, a study using Enzyme-Linked Immunosorbent Assays (ELISA) quantified 200 soluble cytokines, receptors, chemokines, growth factors, and inflammatory factors, and recently demonstrated that the hAMSCs secretome contains molecules associated with the remodeling and homeostasis of the extracellular matrix (ECM) as well as in immunomodulation [13]. Another study, employing ELISA and quantitative PCR, demonstrated the presence of pro-angiogenic factors such as HGF, EGF, and bFGF in the hAMSCs secretome [14]. In addition, a proteomic analysis using in-solution digestion label-free mass spectrometry revealed that the hAMSCs secretome contains proteins associated with wound healing [15]. In particular, the hAMSCs secretome contained high levels of proteins related to angiogenesis, cellular differentiation, immune response, cell motility, and wound healing, such as collagen triple helix repeat-containing protein 1 (CTHRC1), lysyl oxidase homolog 2 (LOXL2), A disintegrin and metalloproteinase with thrombospondin motifs 1 (ADAMTS1), galectin-1 (LGALS1), complement C3 (C3), and CCN family member 1 (CYR61). These proteins are known to promote keratinocyte migration and differentiation [15]. However, a comprehensive characterization of the hAMSCs secretome, capable of providing insights into the vast array of molecules secreted by hAMSCs and potentially implicated in their therapeutic properties, is still lacking. Such characterization would advance our understanding of the molecular mechanisms and the factors contributing to or responsible for the immunomodulatory properties of the hAMSCs secretome. This knowledge could also contribute to the development of new, cell-free therapeutic strategies and the engineering of medical devices for MSC secretome-based treatments in regenerative medicine.

In this study, we characterized the protein content of the hAMSCs secretome using a proteomic approach based on Filter-Aided Sample Preparation (FASP) digestion coupled with Liquid Chromatography-high resolution Mass Spectrometry (LC–MS) analysis. We identified, with high confidence, a protein pattern consisting of 157 elements that consistently characterized the hAMSCs secretome. These elements were further investigated for gene ontology analysis, pathway classification, and relative label-free quantification.

## Methods

### Isolation of mesenchymal stromal cells from human amniotic membrane (hAMSCs)

Human term placentas (*N* = 19) were collected after obtaining written informed consent from mothers after vaginal delivery or cesarean section. The study was conducted in accordance with the Declaration of Helsinki, and informed consent was obtained following the guidelines defined by the Brescia Provincial Ethics Committee (number NP 2243, 19/01/2016).

Amniotic membrane mesenchymal stromal cells (hAMSC) were isolated as previously described [16]. Membrane fragments were digested at 37 °C in dispase (2.5 U/mL, VWR, Radnor, PA, USA) for 9 min and then transferred to RPMI 1640 complete medium containing 10% heat-inactivated fetal bovine serum (FBS), 1% P/S, and 1% L-glutamine (all from Sigma-Aldrich, St. Louis, MO, USA) to block digestion. The fragments were subsequently incubated in collagenase 0.94 mg/mL and DNase I (both from Roche, Basel, Switzerland) for 2.5–3 h at 37 °C. After centrifugation at low g, the resulting supernatant was filtered through a 100-μm cell strainer (BD Falcon, Bedford, MA, USA), and the cells were harvested by centrifugation.

Freshly isolated cells were expanded to passage 1 (p1) by plating them at a density of 10^4^ cells/cm^2^ in Chang Medium C (Irvine Scientific, Santa Ana, CA, USA) supplemented with 2 mM L-glutamine at 37 °C in a 5% CO2 incubator.

hAMSCs at p1 were phenotypically characterized as previously reported [16]. The hAMSCs used in this study met the minimal criteria for consideration as MSCs, namely, the expression of MSC markers CD13 (97.7 ± 1.6%; mean ± SD), CD73 (88.3 ± 6.4%), and CD90 (94.8 ± 7.4%), and the lack of hematopoietic markers such as CD45 (1.8 ± 1.0%), CD66b (0%), and the epithelial marker CD324 (1.7 ± 1.0%) [2, 17, 18].

### Preparation of conditioned medium (CM), lyophilization and reconstitution

hAMSC at p1 were cultured for 5 days in 24-well plates (Corning, NY, USA) at a density of 5 × 10^5^ cells/well in 0.5 mL of DMEM-F12 medium (Sigma-Aldrich) without serum, supplemented with 2 mM L-glutamine (Sigma-Aldrich) and 1% P/S as previously described [1]. After the incubation period, CM was collected, centrifuged at 300 × g, filtered through a 0.2-μm sterile filter (Sartorius Stedim, Florence, Italy) and stored at − 80 °C.

The frozen CM was then lyophilized as previously described [4] using a Lyophilizer Pilot MAX MX 8556 (Millrock Technology, USA), following the previously described procedure [4]. This process involved freezing the samples at − 40 °C per 4 h and then at − 45 °C under vacuum. A first dry cycle, consisting of 7 steps at increasing temperatures was performed for 13 h under vacuum. Subsequently, a second dry cycle was performed under vacuum at 30 °C for one hour. Lyophilization was complete when the product reached 25 °C for at least 1 h. Prior to use, the lyophilized CM was reconstituted with 2.5 mL of sterile water and filtered through a 0.2-μm sterile filter (Sartorius Stedim, Florence, Italy).

A total of 17 placentae were used to produce CM from hAMSC at p1. Each experiment was performed using a mix of CM derived from at least 3 different hAMSC donors, which were previously validated for their immunomodulatory activity, as reported in [4].

### Proteomic analysis

#### Chemicals

All organic solvents were of LC–MS grade. Iodoacetamide (IAA), D,L-dithiothreitol (DTT), ammonium bicarbonate (AMBIC), bovine serum albumin were purchased from Sigma-Aldrich (St. Louis, MO, USA). Water and formic acid (FA) were obtained from Merck (Darmstadt, Germany). Trypsin (Gold MS Grade) was supplied from Promega (Madison, WI, USA), and acetonitrile (ACN) was supplied from Merck (Darmstadt, Germany).

#### Treatment of secretome samples and protein quantification

Four different batches lyophilized hAMSCs secretome (Pools 1–4), with a total volume of 2500 μL each, and three DMEMF12 culture medium samples, used as reference controls (CTRL 1–3), were solubilized in 250 μL of LC–MS grade water to obtain a 10 × concentration. The samples were gently vortexed to facilitate resolubilization. Total protein content was measured using the Bradford protein assay (Bio-Rad Laboratories, Hercules, CA, USA) with a UV–Vis spectrophotometer (8453 UV–Vis Supplies, Agilent Technologies, Waldbronn, Germany), using BSA as the protein of reference.

#### FASP protein digestion protocol

Filter-aided sample preparation (FASP) was employed using centrifugal Microcon filtration devices (Millipore) equipped with a 10 kDa molecular mass cut-off filter membrane for sample purification, concentration, and proteins digestion [19, 20] for a MS-based proteomic analysis. The FASP method provides a higher number of identifications in comparison to in-solution digestion. This makes it a formidable choice for sample preparation in proteomic analysis, especially when dealing with secretomes characterized by a low or diluted protein content. FASP devices, equipped with a molecular cut-off membrane filter, allow efficient protein purification and enzymatic digestion through a series of steps involving buffer exchange, reagent addition, centrifugation, and ultimately protein concentration. This approach proves advantageous for the analysis of conditioned medium.

A secretome volume corresponding to 50 µg of total protein content was mixed with 8 M urea in 0.1 M Tris/HCl buffer at pH 8.5 (Urea Buffer solution), transferred to the filter device, and centrifuged at 14,000 rpm for 15 min. The concentrated sample was diluted in the device with Urea Buffer solution and centrifuged once more. Subsequently, the supernatant was treated with 8 mM DTT in Urea Buffer solution (DTT solution) to reduce disulfide bridges. It was then incubated at 37 °C for 15 min and centrifuged again. Any excess DTT was eliminated through washings with Urea Buffer followed by centrifugations. The supernatant was then treated for thiols carboxamide methylation with 50 mM iodoacetamide (IAA) solution in Urea Buffer. The mixture was incubated in the dark at room temperature (RT) for 15 min, followed by centrifugation. Excess IAA was removed by incubating the sample with DTT solution at 37 °C for 15 min, followed by washes in Urea Buffer solution and then in ammonium bicarbonate for buffer exchange. Sample digestion was carried out overnight at 37 °C using trypsin 1 µg/µL in 1:100 (w/w) in ammonium bicarbonate buffer 50 mM. Enzymatic digestion was stopped by the addition of 1% FA (final concentration). The proteolytic peptides were collected by centrifugation, lyophilized, and then dissolved in 0.1% FA water solution (v/v) for LC–MS analysis.

#### Ultra-high-performance liquid chromatography-nanoESI mass spectrometry analysis (UHPLC-ESI–MS/MS)

UHPLC-ESI–MS/MS analyses were performed for each sample in triplicate on UltiMate 3000 RSLCnano System coupled to Orbitrap Elite MS detector with EASY-Spray nanoESI source (Thermo Fisher Scientific, Waltham, MA, USA). Instrumental operation and data acquisition were performed using Thermo Xcalibur 2.2 computer program (Thermo Fisher Scientific). Each sample was analyzed in replicate chromatographic runs (*n* = 3) to ensure data repeatability, robustness, and the proper operation of the instruments. The proteomic analysis of different batches of the hAMSCs secretome, each with replicate (*n* = 3) LC–MS analyses, along with the parameters applied to software data elaboration and filtering, ensured the repeatability and robustness of protein and peptide identification data.

Chromatographic separations were performed on a PepMap C18 (2 µm particles, 100 Å pore size) EASY-Spray column with a length of 15 cm and an internal diameter (ID) of 50 µm (Thermo Fisher Scientific). This column was coupled to an Acclaim PepMap100 nano-trap cartridge (C18, 5 µm, 100 Å, 300 µm, ID × 5 mm) (Thermo Fisher Scientific). The separation was performed at 40 °C using gradient elution. Eluent A consisted of 0.1% FA, while eluent B was an ACN/FA solution (99.9:0.1, v/v). The gradient elution protocol was as follows: (i) 5% B for 7 min, (ii) 5% to 35% B for 113 min, (iii) 35% B to 99% for 2 min, (iv) 99% B for 3 min, (v) 99% to 1.6% B for 2 min, (vi) 1.6% B for 3 min, (vii) 1.6% to 78% B for 3 min, (viii) 78% B for 3 min, (ix) 78% to 1.6% B for 3 min, (x) 1.6% B for 3 min, (xi) 1.6% to 78% B for 3 min, (xii) 78% B for 3 min, (xiii) 78% B to 5% B for 2 min, and (xiv) 5% B for 20 min. The mobile phase flow rate was 0.3 µL/min. The injection volume was 5 µL. The Orbitrap Elite instrument operated in positive ionization mode with a 60,000 full scan resolution, in 350–2000 m/z acquisition range. MS/MS fragmentation was obtained using collision-induced dissociation (CID) with a normalized collision energy of 35%. The instrument used a Data-Dependent Scan (DDS) mode to perform MS/MS on the 20 most intense signals from each MS spectrum. The minimum signal threshold was set to 500.0, and an isolation width of 2 m/z was applied, with a default charge state to + 2. MS/MS spectra acquisition was performed in the linear ion trap at a normal scan rate.

#### Data analysis

LC–MS and MS/MS raw data were processed using two software tools: the HPLC–MS apparatus management software (Xcalibur 2.0.7 SP1, Thermo Fisher Scientific), and the Proteome Discoverer 1.4 software (version 1.4.1.14, Thermo Fisher Scientific) that was used for protein identification based on the SEQUEST HT cluster as search engine against the Homo Sapiens (UniProtKB/Swiss-Prot protein knowledgebase released in 2021_4), and Bos taurus (UniProtKB/Swiss-Prot protein knowledgebase released in 2022_02). The signal to Noise (S/N) threshold was set to 1.5. Trypsin was used for cleavage with a maximum of 2 missed cleavage sites, and the minimum and maximum peptide length were set to 6 and 144 residues, respectively. Tolerance settings for the analysis included a mass tolerance 10 ppm, fragment mass tolerance of 0.5 Da and 0.02 Da; both “use average precursor mass” and “use average fragment mass” were set to “False”. Methionine oxidation (+ 15.99 Da) and N-Terminal acetylation (+ 42.011 Da) were set as dynamic modifications, while carbamidomethylation of cysteine (+ 57.02 Da) was set as a static modification. Validation of protein and peptide identifications was performed through a decoy database search and calculation of the False Discovery Rate (FDR) statistical value using the Percolator node in Proteome Discoverer workflow. We set strict and relaxed FDR target values at 0.01 and 0.05, respectively. The FDR measures the confidence in the identification by estimating the number of false positive identifications among all the identifications found by a peptide identification search.

The protein identification results from each secretome pool (Pools 1–4) were analyzed as a multireport data file, combining data from three analytical replicates (runs 1–3). The data were filtered to ensure high confidence peptide identification, requiring a minimum of at least 2 peptides per protein, each with a minimum peptide length of 9 amino acids and peptide rank 1, according to the Human Proteome Project (HPP) Mass Spectrometry Data Interpretation Guidelines [21]. Gene ontology (GO) analysis and classification were conducted using Reactome (https://reactome.org) [22] and Protein Analysis Through Evolutionary Relationships (PANTHER, http://www.pantherdb.org) by applying the Fisher’s Exact test type with false discovery rate (FDR) correction for statistical test of over-representation [23]. The protein expression data were sourced from The Human Protein Atlas (https://www.proteinatlas.org) [24, 25]. Functional protein interaction networks were analyzed using the STRING tool [26] with the highest confidence level (0.900), and Cytoscape (https://cytoscape.org/). Sample data grouping analysis was performed using the Venn diagram tool (https://bioinfogp.cnb.csic.es/tools/venny).

## Results

The proteomic profiles of four different hAMSCs-CM samples obtained from serum-free cell cultures were characterized using LC–MS analysis, after Filter-Aided Sample Preparation (FASP) digestion with trypsin. To identify common protein elements in comparison to a cell-free DMEMF12 culture medium used as a control, the data obtained for each pool were subjected to grouping analysis. The resulting list of proteins, which consistently characterized the hAMSCs secretome, and could thus be associated with its biological activities, was obtained by Proteome Discoverer data elaboration after validation by the Percolator node and FDR statistical value calculation and filtered for high confidence identification according to the Human Proteome Project Mass Spectrometry Data Interpretation Guidelines [21]. Bioinformatics tools were applied to investigate the gene ontology classification, pathway over-representation, and protein functional interactions of the identified proteins.

### Proteomic characterization of the hAMSCs secretome

The total protein content of each hAMSC-CM pool was determined using the Bradford assay. The mean value for the 4 pools was 0.49 ± 0.23 mg/ml. This measurement was obtained after re-dissolving the lyophilized pools to a 10 × concentration in water. For proteomic analysis, an aliquot of the each pool, equivalent to 50 μg of total protein, underwent FASP digestion and LC–MS analysis in triplicate runs for proteomic characterization.

**Fig. 1:**
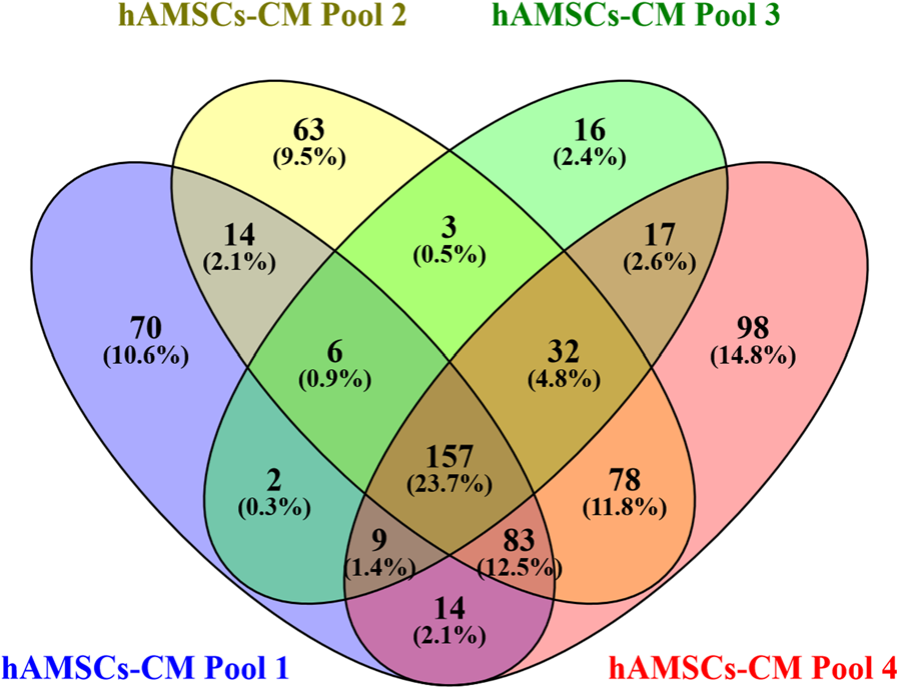
Venn diagram resulting from grouping analysis of the proteins identified in the hAMSCs-CM pools 1–4 (list of identifications per pool in Additional file 1: Table S1)

The Proteome Discoverer software processed the LC–MS data and identified a total of 1521 proteins in the hAMSCs secretome with a high level of confidence. These proteins were distributed differently among the analyzed pools, with 355, 436, 242, and 488 proteins identified in pools 1 through 4, respectively (protein identification data in Additional file 1: Table S1). Grouping analysis revealed a total of 662 unique elements with 157 proteins shared by all the hAMSCs-CM pools (Fig. 1 and Table 1). These 157 proteins, corresponding to 23.7%, of the total unique elements, characterize the hAMSCs secretome and thus may be associated to the biological activities exerted by hAMSCs-CM.

**Table 1:** List of the 157 proteins elements commonly identified in hAMSCs secretome pools 1–4

Uniprot accession	Protein name	Gene name	MW [kDa]
P62328	Thymosin beta-4	TYB4	5,0
P02795	Metallothionein-2	MT2	6,0
P05387	60S acidic ribosomal protein P2	RLA2	11,7
P99999	Cytochrome c	CYC	11,7
O75368	SH3 domain-binding glutamic acid-rich-like protein	SH3L1	12,8
P09382	Galectin-1	LEG1	14,7
P0DP25	Calmodulin-3	CALM3	16,8
P60660	Myosin light polypeptide 6	MYL6	16,9
P15531	Nucleoside diphosphate kinase A	NDKA	17,1
P62937	Peptidyl-prolyl cis–trans isomerase A	PPIA	18,0
P19105	Myosin regulatory light chain 12A	ML12A	19,8
Q99497	Parkinson disease protein 7	PARK7	19,9
P21291	Cysteine and glycine-rich protein 1	CSRP1	20,6
P55145	Mesencephalic astrocyte-derived neurotrophic factor	MANF	20,7
Q06830	Peroxiredoxin-1	PRDX1	22,1
P37802	Transgelin-2	TAGL2	22,4
Q01995	Transgelin	TAGL	22,6
P80723	Brain acid soluble protein 1	BASP1	22,7
P01033	Metalloproteinase inhibitor 1	TIMP1	23,2
P09211	Glutathione S-transferase P	GSTP1	23,3
P23284	Peptidyl-prolyl cis–trans isomerase B	PPIB	23,7
P16035	Metalloproteinase inhibitor 2	TIMP2	24,4
P09936	Ubiquitin carboxyl-terminal hydrolase isozyme L1	UCHL1	24,8
P62906	60S ribosomal protein L10a	RL10A	24,8
P20618	Proteasome subunit beta type-1	PSB1	26,5
P60174	Triosephosphate isomerase	TPIS	26,7
P48307	Tissue factor pathway inhibitor 2	TFPI2	26,9
P78417	Glutathione S-transferase omega-1	GSTO1	27,5
P52823	Stanniocalcin-1	STC1	27,6
P63104	14–3-3 protein zeta/delta	1433Z	27,7
P61981	14–3-3 protein gamma	1433G	28,3
P25788	Proteasome subunit alpha type-3	PSA3	28,4
P67936	Tropomyosin alpha-4 chain	TPM4	28,5
Q16270	Insulin-like growth factor-binding protein 7	IBP7	29,1
P62258	14–3-3 protein epsilon	1433E	29,2
P28070	Proteasome subunit beta type-4	PSB4	29,2
O00584	Ribonuclease T2	RNT2	29,5
P25786	Proteasome subunit alpha type-1	PSA1	29,5
P47756	F-actin-capping protein subunit beta	CAPZB	31,3
P29966	Myristoylated alanine-rich C-kinase substrate	MARCS	31,5
P17936	Insulin-like growth factor-binding protein 3	IBP3	31,7
P00491	Purine nucleoside phosphorylase	PNPH	32,1
P09486	SPARC	SPRC	34,6
Q12841	Follistatin-related protein 1	FSTL1	35,0
P40926	Malate dehydrogenase, mitochondrial	MDHM	35,5
P08758	Annexin A5	ANXA5	35,9
P04406	Glyceraldehyde-3-phosphate dehydrogenase	G3P	36,0
P40925	Malate dehydrogenase, cytoplasmic	MDHC	36,4
P07195	L-lactate dehydrogenase B chain	LDHB	36,6
P00338	L-lactate dehydrogenase A chain	LDHA	36,7
O43852	Calumenin	CALU	37,1
P37837	Transaldolase	TALDO	37,5
P07858	Cathepsin B	CATB	37,8
Q9UBP4	Dickkopf-related protein 3	DKK3	38,4
P07355	Annexin A2	ANXA2	38,6
P04083	Annexin A1	ANXA1	38,7
Q15293	Reticulocalbin-1	RCN1	38,9
P04075	Fructose-bisphosphate aldolase A	ALDOA	39,4
P60709	Actin, cytoplasmic 1	ACTB	41,7
P36222	Chitinase-3-like protein 1	CH3L1	42,6
P07093	Glia-derived nexin	GDN	44,0
P08727	Keratin, type I cytoskeletal 19	K1C19	44,1
P07339	Cathepsin D	CATD	44,5
P00558	Phosphoglycerate kinase 1	PGK1	44,6
P05121	Plasminogen activator inhibitor 1	PAI1	45,0
P50454	Serpin H1	SERPH	46,4
P06733	Alpha-enolase	ENOA	47,1
Q8NBS9	Thioredoxin domain-containing protein 5	TXND5	47,6
Q15113	Procollagen C-endopeptidase enhancer 1	PCOC1	47,9
Q15084	Protein disulfide-isomerase A6	PDIA6	48,1
P27797	Calreticulin	CALR	48,1
O95967	EGF-containing fibulin-like extracellular matrix protein 2	FBLN4	49,4
P13489	Ribonuclease inhibitor	RINI	49,9
P26641	Elongation factor 1-gamma	EF1G	50,1
P68363	Tubulin alpha-1B chain	TBA1B	50,1
P31150	Rab GDP dissociation inhibitor alpha	GDIA	50,6
P50395	Rab GDP dissociation inhibitor beta	GDIB	50,6
Q01518	Adenylyl cyclase-associated protein 1	CAP1	51,9
P05787	Keratin, type II cytoskeletal 8	K2C8	53,7
P03956	Interstitial collagenase	MMP1	54,0
P09238	Stromelysin-2	MMP10	54,1
P30101	Protein disulfide-isomerase A3	PDIA3	56,7
Q16851	UTP–glucose-1-phosphate uridylyltransferase	UGPA	56,9
P07237	Protein disulfide-isomerase	PDIA1	57,1
P12081	Histidine–tRNA ligase, cytoplasmic	HARS1	57,4
P14618	Pyruvate kinase PKM	KPYM	57,9
P07602	Prosaposin	SAP	58,1
P13645	Keratin, type I cytoskeletal 10	K1C10	58,8
P14314	Glucosidase 2 subunit beta	GLU2B	59,4
P35527	Keratin, type I cytoskeletal 9	K1C9	62,0
P06744	Glucose-6-phosphate isomerase	G6PI	63,1
P14866	Heterogeneous nuclear ribonucleoprotein L	HNRPL	64,1
Q08380	Galectin-3-binding protein	LG3BP	65,3
P35908	Keratin, type II cytoskeletal 2 epidermal	K22E	65,4
P04264	Keratin, type II cytoskeletal 1	K2C1	66,0
P29401	Transketolase	TKT	67,8
P02768	Albumin	ALBU	69,3
P0DMV8	Heat shock 70 kDa protein 1A	HS71A	70,0
P13797	Plastin-3	PLST	70,8
P11142	Heat shock cognate 71 kDa protein	HSP7C	70,9
Q16881	Thioredoxin reductase 1, cytoplasmic	TRXR1	70,9
P11021	Endoplasmic reticulum chaperone BiP	BIP	72,3
P13667	Protein disulfide-isomerase A4	PDIA4	72,9
P08253	72 kDa type IV collagenase	MMP2	73,8
P02545	Prelamin-A/C	LMNA	74,1
Q15582	Transforming growth factor-beta-induced protein ig-h3	BGH3	74,6
P09871	Complement C1s subcomponent	C1S	76,6
P02787	Serotransferrin	TRFE	77,0
P00736	Complement C1r subcomponent	C1R	80,1
O00391	Sulfhydryl oxidase 1	QSOX1	82,5
P08238	Heat shock protein HSP 90-beta	HS90B	83,2
O00469	Procollagen-lysine,2-oxoglutarate 5-dioxygenase 2	PLOD2	84,6
P06396	Gelsolin	GELS	85,6
Q9Y4K0	Lysyl oxidase homolog 2	LOXL2	86,7
P05556	Integrin beta-1	ITB1	88,4
P55072	Transitional endoplasmic reticulum ATPase	TERA	89,3
P14625	Endoplasmin	ENPL	92,4
Q15063	Periostin	POSTN	93,3
P13639	Elongation factor 2	EF2	95,3
Q14764	Major vault protein	MVP	99,3
P12814	Alpha-actinin-1	ACTN1	103,0
P55786	Puromycin-sensitive aminopeptidase	PSA	103,2
O43707	Alpha-actinin-4	ACTN4	104,8
P10253	Lysosomal alpha-glucosidase	LYAG	105,3
Q9Y6C2	EMILIN-1	EMIL1	106,6
P12109	Collagen alpha-1(VI) chain	CO6A1	108,5
P15144	Aminopeptidase N	AMPN	109,5
P27816	Microtubule-associated protein 4	MAP4	120,9
P18206	Vinculin	VINC	123,7
P08123	Collagen alpha-2(I) chain	CO1A2	129,2
P07996	Thrombospondin-1	TSP1	129,3
P35442	Thrombospondin-2	TSP2	129,9
P02461	Collagen alpha-1(III) chain	CO3A1	138,5
P02452	Collagen alpha-1(I) chain	CO1A1	138,9
Q14112	Nidogen-2	NID2	151,2
P08572	Collagen alpha-2(IV) chain	CO4A2	167,4
P11047	Laminin subunit gamma-1	LAMC1	177,5
Q13219	Pappalysin-1	PAPP1	180,9
P20908	Collagen alpha-1(V) chain	CO5A1	183,4
Q14766	Latent-transforming growth factor beta-binding protein 1	LTBP1	186,7
P01024	Complement C3	CO3	187,0
P46940	Ras GTPase-activating-like protein IQGAP1	IQGA1	189,1
Q14767	Latent-transforming growth factor beta-binding protein 2	LTBP2	194,9
P07942	Laminin subunit beta-1	LAMB1	197,9
P35579	Myosin-9	MYH9	226,4
Q9Y490	Talin-1	TLN1	269,6
P02751	Fibronectin	FINC	272,2
O75369	Filamin-B	FLNB	278,0
P21333	Filamin-A	FLNA	280,6
Q13813	Spectrin alpha chain, non-erythrocytic 1	SPTN1	284,4
Q14315	Filamin-C	FLNC	290,8
P35555	Fibrillin-1	FBN1	312,1
Q99715	Collagen alpha-1(XII) chain	COCA1	332,9
P12111	Collagen alpha-3(VI) chain	CO6A3	343,5
P13611	Versican core protein	CSPG2	372,6
P98160	Basement membrane-specific heparan sulfate proteoglycan core protein	PGBM	468,5
Q07954	Prolow-density lipoprotein receptor-related protein 1	LRP1	504,3
P62328	Thymosin beta-4	TYB4	5,0
P02795	Metallothionein-2	MT2	6,0
P05387	60S acidic ribosomal protein P2	RLA2	11,7
P99999	Cytochrome c	CYC	11,7
O75368	SH3 domain-binding glutamic acid-rich-like protein	SH3L1	12,8
P09382	Galectin-1	LEG1	14,7
P0DP25	Calmodulin-3	CALM3	16,8
P60660	Myosin light polypeptide 6	MYL6	16,9
P15531	Nucleoside diphosphate kinase A	NDKA	17,1
P62937	Peptidyl-prolyl cis–trans isomerase A	PPIA	18,0
P19105	Myosin regulatory light chain 12A	ML12A	19,8
Q99497	Parkinson disease protein 7	PARK7	19,9
P21291	Cysteine and glycine-rich protein 1	CSRP1	20,6
P55145	Mesencephalic astrocyte-derived neurotrophic factor	MANF	20,7
Q06830	Peroxiredoxin-1	PRDX1	22,1
P37802	Transgelin-2	TAGL2	22,4
Q01995	Transgelin	TAGL	22,6
P80723	Brain acid soluble protein 1	BASP1	22,7
P01033	Metalloproteinase inhibitor 1	TIMP1	23,2
P09211	Glutathione S-transferase P	GSTP1	23,3
P23284	Peptidyl-prolyl cis–trans isomerase B	PPIB	23,7
P16035	Metalloproteinase inhibitor 2	TIMP2	24,4
P09936	Ubiquitin carboxyl-terminal hydrolase isozyme L1	UCHL1	24,8
P62906	60S ribosomal protein L10a	RL10A	24,8
P20618	Proteasome subunit beta type-1	PSB1	26,5
P60174	Triosephosphate isomerase	TPIS	26,7
P48307	Tissue factor pathway inhibitor 2	TFPI2	26,9
P78417	Glutathione S-transferase omega-1	GSTO1	27,5
P52823	Stanniocalcin-1	STC1	27,6
P63104	14–3-3 protein zeta/delta	1433Z	27,7
P61981	14–3-3 protein gamma	1433G	28,3
P25788	Proteasome subunit alpha type-3	PSA3	28,4
P67936	Tropomyosin alpha-4 chain	TPM4	28,5
Q16270	Insulin-like growth factor-binding protein 7	IBP7	29,1
P62258	14–3-3 protein epsilon	1433E	29,2
P28070	Proteasome subunit beta type-4	PSB4	29,2
O00584	Ribonuclease T2	RNT2	29,5
P25786	Proteasome subunit alpha type-1	PSA1	29,5
P47756	F-actin-capping protein subunit beta	CAPZB	31,3
P29966	Myristoylated alanine-rich C-kinase substrate	MARCS	31,5
P17936	Insulin-like growth factor-binding protein 3	IBP3	31,7
P00491	Purine nucleoside phosphorylase	PNPH	32,1
P09486	SPARC	SPRC	34,6
Q12841	Follistatin-related protein 1	FSTL1	35,0
P40926	Malate dehydrogenase, mitochondrial	MDHM	35,5
P08758	Annexin A5	ANXA5	35,9
P04406	Glyceraldehyde-3-phosphate dehydrogenase	G3P	36,0
P40925	Malate dehydrogenase, cytoplasmic	MDHC	36,4
P07195	L-lactate dehydrogenase B chain	LDHB	36,6
P00338	L-lactate dehydrogenase A chain	LDHA	36,7
O43852	Calumenin	CALU	37,1
P37837	Transaldolase	TALDO	37,5
P07858	Cathepsin B	CATB	37,8
Q9UBP4	Dickkopf-related protein 3	DKK3	38,4
P07355	Annexin A2	ANXA2	38,6
P04083	Annexin A1	ANXA1	38,7
Q15293	Reticulocalbin-1	RCN1	38,9
P04075	Fructose-bisphosphate aldolase A	ALDOA	39,4
P60709	Actin, cytoplasmic 1	ACTB	41,7
P36222	Chitinase-3-like protein 1	CH3L1	42,6
P07093	Glia-derived nexin	GDN	44,0
P08727	Keratin, type I cytoskeletal 19	K1C19	44,1
P07339	Cathepsin D	CATD	44,5
P00558	Phosphoglycerate kinase 1	PGK1	44,6
P05121	Plasminogen activator inhibitor 1	PAI1	45,0
P50454	Serpin H1	SERPH	46,4
P06733	Alpha-enolase	ENOA	47,1
Q8NBS9	Thioredoxin domain-containing protein 5	TXND5	47,6
Q15113	Procollagen C-endopeptidase enhancer 1	PCOC1	47,9
Q15084	Protein disulfide-isomerase A6	PDIA6	48,1
P27797	Calreticulin	CALR	48,1
O95967	EGF-containing fibulin-like extracellular matrix protein 2	FBLN4	49,4
P13489	Ribonuclease inhibitor	RINI	49,9
P26641	Elongation factor 1-gamma	EF1G	50,1
P68363	Tubulin alpha-1B chain	TBA1B	50,1
P31150	Rab GDP dissociation inhibitor alpha	GDIA	50,6
P50395	Rab GDP dissociation inhibitor beta	GDIB	50,6
Q01518	Adenylyl cyclase-associated protein 1	CAP1	51,9
P05787	Keratin, type II cytoskeletal 8	K2C8	53,7
P03956	Interstitial collagenase	MMP1	54,0
P09238	Stromelysin-2	MMP10	54,1
P30101	Protein disulfide-isomerase A3	PDIA3	56,7
Q16851	UTP–glucose-1-phosphate uridylyltransferase	UGPA	56,9
P07237	Protein disulfide-isomerase	PDIA1	57,1
P12081	Histidine–tRNA ligase, cytoplasmic	HARS1	57,4
P14618	Pyruvate kinase PKM	KPYM	57,9
P07602	Prosaposin	SAP	58,1
P13645	Keratin, type I cytoskeletal 10	K1C10	58,8
P14314	Glucosidase 2 subunit beta	GLU2B	59,4
P35527	Keratin, type I cytoskeletal 9	K1C9	62,0
P06744	Glucose-6-phosphate isomerase	G6PI	63,1
P14866	Heterogeneous nuclear ribonucleoprotein L	HNRPL	64,1
Q08380	Galectin-3-binding protein	LG3BP	65,3
P35908	Keratin, type II cytoskeletal 2 epidermal	K22E	65,4
P04264	Keratin, type II cytoskeletal 1	K2C1	66,0
P29401	Transketolase	TKT	67,8
P02768	Albumin	ALBU	69,3
P0DMV8	Heat shock 70 kDa protein 1A	HS71A	70,0
P13797	Plastin-3	PLST	70,8
P11142	Heat shock cognate 71 kDa protein	HSP7C	70,9
Q16881	Thioredoxin reductase 1, cytoplasmic	TRXR1	70,9
P11021	Endoplasmic reticulum chaperone BiP	BIP	72,3
P13667	Protein disulfide-isomerase A4	PDIA4	72,9
P08253	72 kDa type IV collagenase	MMP2	73,8
P02545	Prelamin-A/C	LMNA	74,1
Q15582	Transforming growth factor-beta-induced protein ig-h3	BGH3	74,6
P09871	Complement C1s subcomponent	C1S	76,6
P02787	Serotransferrin	TRFE	77,0
P00736	Complement C1r subcomponent	C1R	80,1
O00391	Sulfhydryl oxidase 1	QSOX1	82,5
P08238	Heat shock protein HSP 90-beta	HS90B	83,2
O00469	Procollagen-lysine,2-oxoglutarate 5-dioxygenase 2	PLOD2	84,6
P06396	Gelsolin	GELS	85,6
Q9Y4K0	Lysyl oxidase homolog 2	LOXL2	86,7
P05556	Integrin beta-1	ITB1	88,4
P55072	Transitional endoplasmic reticulum ATPase	TERA	89,3
P14625	Endoplasmin	ENPL	92,4
Q15063	Periostin	POSTN	93,3
P13639	Elongation factor 2	EF2	95,3
Q14764	Major vault protein	MVP	99,3
P12814	Alpha-actinin-1	ACTN1	103,0
P55786	Puromycin-sensitive aminopeptidase	PSA	103,2
O43707	Alpha-actinin-4	ACTN4	104,8
P10253	Lysosomal alpha-glucosidase	LYAG	105,3
Q9Y6C2	EMILIN-1	EMIL1	106,6
P12109	Collagen alpha-1(VI) chain	CO6A1	108,5
P15144	Aminopeptidase N	AMPN	109,5
P27816	Microtubule-associated protein 4	MAP4	120,9
P18206	Vinculin	VINC	123,7
P08123	Collagen alpha-2(I) chain	CO1A2	129,2
P07996	Thrombospondin-1	TSP1	129,3
P35442	Thrombospondin-2	TSP2	129,9
P02461	Collagen alpha-1(III) chain	CO3A1	138,5
P02452	Collagen alpha-1(I) chain	CO1A1	138,9
Q14112	Nidogen-2	NID2	151,2
P08572	Collagen alpha-2(IV) chain	CO4A2	167,4
P11047	Laminin subunit gamma-1	LAMC1	177,5
Q13219	Pappalysin-1	PAPP1	180,9
P20908	Collagen alpha-1(V) chain	CO5A1	183,4
Q14766	Latent-transforming growth factor beta-binding protein 1	LTBP1	186,7
P01024	Complement C3	CO3	187,0
P46940	Ras GTPase-activating-like protein IQGAP1	IQGA1	189,1
Q14767	Latent-transforming growth factor beta-binding protein 2	LTBP2	194,9
P07942	Laminin subunit beta-1	LAMB1	197,9
P35579	Myosin-9	MYH9	226,4
Q9Y490	Talin-1	TLN1	269,6
P02751	Fibronectin	FINC	272,2
O75369	Filamin-B	FLNB	278,0
P21333	Filamin-A	FLNA	280,6
Q13813	Spectrin alpha chain, non-erythrocytic 1	SPTN1	284,4
Q14315	Filamin-C	FLNC	290,8
P35555	Fibrillin-1	FBN1	312,1
Q99715	Collagen alpha-1(XII) chain	COCA1	332,9
P12111	Collagen alpha-3(VI) chain	CO6A3	343,5
P13611	Versican core protein	CSPG2	372,6
P98160	Basement membrane-specific heparan sulfate proteoglycan core protein	PGBM	468,5
Q07954	Prolow-density lipoprotein receptor-related protein 1	LRP1	504,3

As shown in Table 1, the 157 proteins shared by all hAMSCs-CM pools exhibited a wide range of molecular masses, spanning from 5 to 504 kDa. Interestingly, despite the cut-off of the FASP membrane filter, thymosin beta 4 and metallothionein 2, both with molecular masses < 10 kDa, were identified. One possible explanation could be the existence of protein complexes or dimeric peptides in hAMSCs-CM. Such complexes or dimers could account for their retention by the filter. Indeed, several reports have demonstrated the presence of actin-profilin complexes involving thymosin beta 4, the main G-actin sequestering agent, as well as the oligomerization of metallothioneins [27, 28], which could support our hypothesis.

The same proteomic analysis was applied to three different samples of cell free DMEMF12 culture medium, used as reference control samples (CTRL 1–3), in order to evaluate the influence of the medium’s composition on the proteome composition of hAMSCs-CM. Specifically for CTRL samples, protein identification was obtained by elaborating the LC–MS data against both the *Homo sapiens* and the *Bos taurus* protein databases (protein identification data of CTRL samples 1–3 are in Additional file 2: Table S2A-F). Additional file 2: Table S2G lists the proteins consistently identified in all DMEMF12 samples analyzed, therefore characterizing with repeatability the culture medium matrix, and representing the putative contaminants to be excluded from the analysis of proteins originating from the secretome. This list includes common contaminants such as keratins and albumin, which are frequently detected in proteomic analyses. Keratins are often associated with dust/contact-related contaminants, while albumin can derive from reagents and materials during the sample preparation step [29–31].

Since albumin was part of the 157 common protein elements of the hAMSCs secretome, we compared the pattern of the relative tryptic peptides identified either in the hAMSCs secretome or in DMEMF12, with both the *Homo sapiens* and *Bos taurus* databases (Additional file 2: Table S2H). The absence of peptides exclusively belonging to human albumin in the hAMSCs secretome suggests that the protein or its fragment peptides are probable contaminants rather than components of the secretome. Given the serum-free nature of the medium, it can be concluded that the contribution of the DMEMF12 to the proteome of the hAMSCs secretome is negligible.

### Gene ontology analysis of the 157 proteins characterizing the hAMSCs secretome

Gene ontology classification and over-representation analysis of the molecular function, cellular component, and protein class of the 157 proteins which repeatedly characterize the hAMSCs secretome were performed using PANTHER (www.pantherdb.org, accessed on August 23, 2022). Among the 157 proteins, different molecular functions were found to be significantly over- or under-represented (p value < 0.05, Fisher’s Exact statistical test type with FDR correction) when compared to the reference classification *Homo sapiens* (Table 2). The fold enrichment values revealed that the over-represented molecular functions with the highest values were disulfide isomerase activity, collagen and extracellular matrix binding, and disulfide oxidoreductase activity.

**Table 2:** List of the molecular functions with statistically significant over-/under-representation in the group of the 157 common proteins of hAMSCs secretome pools 1–4

Molecular function	Homo sapiens REFLIST (20,589)	Client text box input (157)	Client text box input (expected)	Client text box input (over/ under)	Client text box input (fold Enrichment)	Client text box input (raw *P*-value)	Client text box input (FDR)
Protein disulfide isomerase activity (GO:0003756)	10	5	0.08	+	65.57	6.58E-08	3.59E-05
Collagen binding (GO:0005518)	8	2	0.06	+	32.79	2.46E-03	2.44E-02
Extracellular matrix binding (GO:0050840)	13	3	0.1	+	30.26	2.21E-04	4.03E-03
Disulfide oxidoreductase activity (GO:0015036)	18	4	0.14	+	29.14	2.08E-05	7.56E-04
Protein-disulfide reductase activity (GO:0015035)	15	3	0.11	+	26.23	3.19E-04	4.84E-03
Misfolded protein binding (GO:0051787)	16	3	0.12	+	24.59	3.77E-04	5.42E-03
Aminopeptidase activity (GO:0004177)	11	2	0.08	+	23.84	4.20E-03	3.76E-02
Oxidoreductase activity, acting on a sulfur group of donors (GO:0016667)	26	4	0.2	+	20.18	7.42E-05	1.84E-03
ATP binding (GO:0005524)	41	5	0.31	+	15.99	2.48E-05	7.98E-04
Isomerase activity (GO:0016853)	76	8	0.58	+	13.8	2.41E-07	6.58E-05
Serine-type endopeptidase inhibitor activity (GO:0004867)	40	4	0.31	+	13.11	3.39E-04	5.00E-03
Endopeptidase inhibitor activity (GO:0004866)	66	6	0.5	+	11.92	1.77E-05	7.42E-04
Heat shock protein binding (GO:0031072)	33	3	0.25	+	11.92	2.53E-03	2.46E-02
Peptidase inhibitor activity (GO:0030414)	66	6	0.5	+	11.92	1.77E-05	6.89E-04
Endopeptidase regulator activity (GO:0061135)	70	6	0.53	+	11.24	2.41E-05	8.23E-04
Peptidase regulator activity (GO:0061134)	72	6	0.55	+	10.93	2.80E-05	8.48E-04
Protease binding (GO:0002020)	78	6	0.59	+	10.09	4.26E-05	1.11E-03
Unfolded protein binding (GO:0051082)	69	5	0.53	+	9.5	2.46E-04	4.34E-03
Actin filament binding (GO:0051015)	135	9	1.03	+	8.74	1.54E-06	1.40E-04
Enzyme inhibitor activity (GO:0004857)	115	7	0.88	+	7.98	4.02E-05	1.16E-03
Metallopeptidase activity (GO:0008237)	116	7	0.88	+	7.91	4.24E-05	1.16E-03
Metalloendopeptidase activity (GO:0004222)	84	5	0.64	+	7.81	5.80E-04	7.19E-03
Serine-type endopeptidase activity (GO:0004252)	103	6	0.79	+	7.64	1.82E-04	3.68E-03
Serine hydrolase activity (GO:0017171)	107	6	0.82	+	7.35	2.21E-04	4.32E-03
Serine-type peptidase activity (GO:0008236)	107	6	0.82	+	7.35	2.21E-04	4.17E-03
Actin binding (GO:0003779)	199	11	1.52	+	7.25	6.06E-07	6.61E-05
Calcium ion binding (GO:0005509)	168	9	1.28	+	7.03	8.38E-06	5.08E-04
Cell adhesion molecule binding (GO:0050839)	141	6	1.08	+	5.58	8.98E-04	1.07E-02
Metal ion binding (GO:0046872)	268	11	2.04	+	5.38	9.44E-06	5.15E-04
Protein-containing complex binding (GO:0044877)	394	15	3	+	4.99	5.34E-07	7.28E-05
Endopeptidase activity (GO:0004175)	324	12	2.47	+	4.86	9.99E-06	4.96E-04
Cation binding (GO:0043169)	325	12	2.48	+	4.84	1.03E-05	4.68E-04
Peptidase activity (GO:0008233)	433	15	3.3	+	4.54	1.66E-06	1.30E-04
Cytoskeletal protein binding (GO:0008092)	411	14	3.13	+	4.47	4.52E-06	3.09E-04
Purine ribonucleotide binding (GO:0032555)	271	8	2.07	+	3.87	1.32E-03	1.47E-02
Carbohydrate derivative binding (GO:0097367)	345	10	2.63	+	3.8	3.84E-04	5.38E-03
Ribonucleotide binding (GO:0032553)	278	8	2.12	+	3.77	1.54E-03	1.68E-02
Ion binding (GO:0043167)	741	21	5.65	+	3.72	3.17E-07	5.77E-05
Purine nucleotide binding (GO:0017076)	288	8	2.2	+	3.64	1.91E-03	2.04E-02
Nucleoside phosphate binding (GO:1,901,265)	330	8	2.52	+	3.18	4.29E-03	3.78E-02
Nucleotide binding (GO:0000166)	330	8	2.52	+	3.18	4.29E-03	3.72E-02
Oxidoreductase activity (GO:0016491)	431	10	3.29	+	3.04	1.99E-03	2.09E-02
Anion binding (GO:0043168)	437	10	3.33	+	3	2.19E-03	2.26E-02
Small molecule binding (GO:0036094)	440	10	3.36	+	2.98	2.30E-03	2.33E-02
Enzyme regulator activity (GO:0030234)	472	10	3.6	+	2.78	3.75E-03	3.42E-02
Catalytic activity, acting on a protein (GO:0140096)	1473	25	11.23	+	2.23	2.50E-04	4.27E-03
Hydrolase activity (GO:0016787)	1739	26	13.26	+	1.96	1.29E-03	1.46E-02
Protein binding (GO:0005515)	2812	40	21.44	+	1.87	9.45E-05	2.15E-03
Catalytic activity (GO:0003824)	3916	50	29.86	+	1.67	1.38E-04	3.02E-03
Nucleic acid binding (GO:0003676)	1974	3	15.05	−	0.2	2.95E-04	4.60E-03
Molecular transducer activity (GO:0060089)	1085	1	8.27	−	0.12	3.51E-03	3.31E-02
Signaling receptor activity (GO:0038023)	1085	1	8.27	−	0.12	3.51E-03	3.25E-02
DNA binding (GO:0003677)	1361	1	10.38	−	0.1	5.15E-04	6.53E-03
Transporter activity (GO:0005215)	770	0	5.87	−	< 0.01	4.76E-03	4.06E-02
Transcription regulator activity (GO:0140110)	1265	0	9.65	−	< 0.01	7.79E-05	1.85E-03
cis-regulatory region sequence-specific DNA binding (GO:0000987)	810	0	6.18	−	< 0.01	3.15E-03	3.02E-02
DNA-binding transcription factor activity, RNA polymerase II-specific (GO:0000981)	984	0	7.5	−	< 0.01	9.51E-04	1.10E-02
RNA polymerase II cis-regulatory region sequence-specific DNA binding (GO:0000978)	798	0	6.09	−	< 0.01	5.09E-03	4.27E-02
RNA polymerase II transcription regulatory region sequence-specific DNA binding (GO:0000977)	1059	0	8.08	−	< 0.01	4.09E-04	5.59E-03
Transcription cis-regulatory region binding (GO:0000976)	1099	0	8.38	−	< 0.01	4.33E-04	5.77E-03
Double-stranded DNA binding (GO:0003690)	1164	0	8.88	−	< 0.01	1.79E-04	3.76E-03
Sequence-specific DNA binding (GO:0043565)	1148	0	8.75	−	< 0.01	2.85E-04	4.57E-03
DNA-binding transcription factor activity (GO:0003700)	1054	0	8.04	−	< 0.01	6.67E-04	8.09E-03
Sequence-specific double-stranded DNA binding (GO:1,990,837)	1117	0	8.52	−	< 0.01	2.71E-04	4.48E-03
Transcription regulatory region nucleic acid binding (GO:0001067)	1099	0	8.38	−	< 0.01	4.33E-04	5.63E-03

**Fig. 2:**
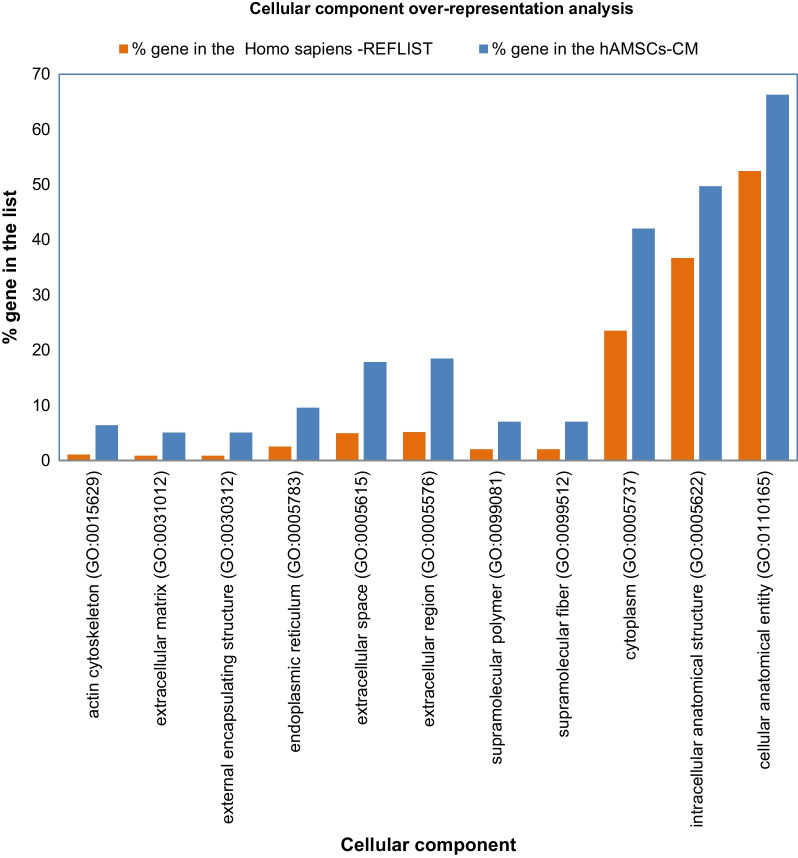
Protein cellular component over-representation analysis of the157 proteins commonly identified in hAMSC-CM using *Homo sapiens* database as reference (Over-representation Test PANTHER GO-Slim Cellular Component, FISHER’s Exact Test Type with FDR correction, *P* < 0.05)

Figure 2 shows the results of the cellular components overrepresentation analysis (*p* value < 0.05, Fisher’s Exact test type with FDR correction), conducted using the 157 proteins characterizing the hAMSCs secretome (blue histograms), in comparison with the *Homo sapiens* list of genes used as a reference (orange histograms). While statistically significant, the fold enrichment values for cellular components were not as high as those observed for molecular functions, actin cytoskeleton, extracellular matrix, external encapsulating structure, endoplasmic reticulum, extracellular space and extracellular region cellular components, which showed the highest values.

**Fig. 3:**
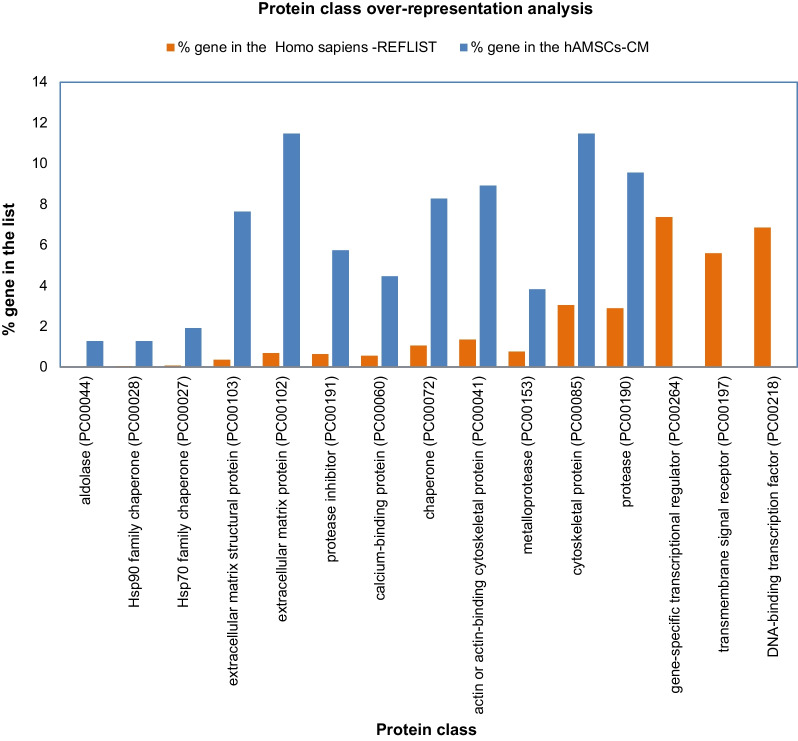
Protein class over-representation analysis of the157 proteins commonly identified in hAMSC-CM using Homo sapiens database as reference (Over-representation Test PANTHER GO-Slim, FISHER’s Exact Test Type with FDR correction, *P* < 0.05)

Relative to the identified proteins in hAMSCs secretome pools, the highest % of genes belonged to extracellular matrix and structural proteins, chaperons, proteases, cytoskeletal proteins, and metalloproteases. This aligns with the biological activity of the hAMSCs secretome exerted during the proliferative and the remodeling phases of the healing process [32]. The aldolase protein class, followed by the Hsp90 family and Hsp70 family chaperones, as well as extracellular matrix proteins, showed the highest fold enrichment values. Conversely, the protein classes of gene-specific transcriptional regulator, transmembrane signal receptor, and DNA-binding transcription factor resulted instead under-represented (Fig. 3).

**Fig. 4:**
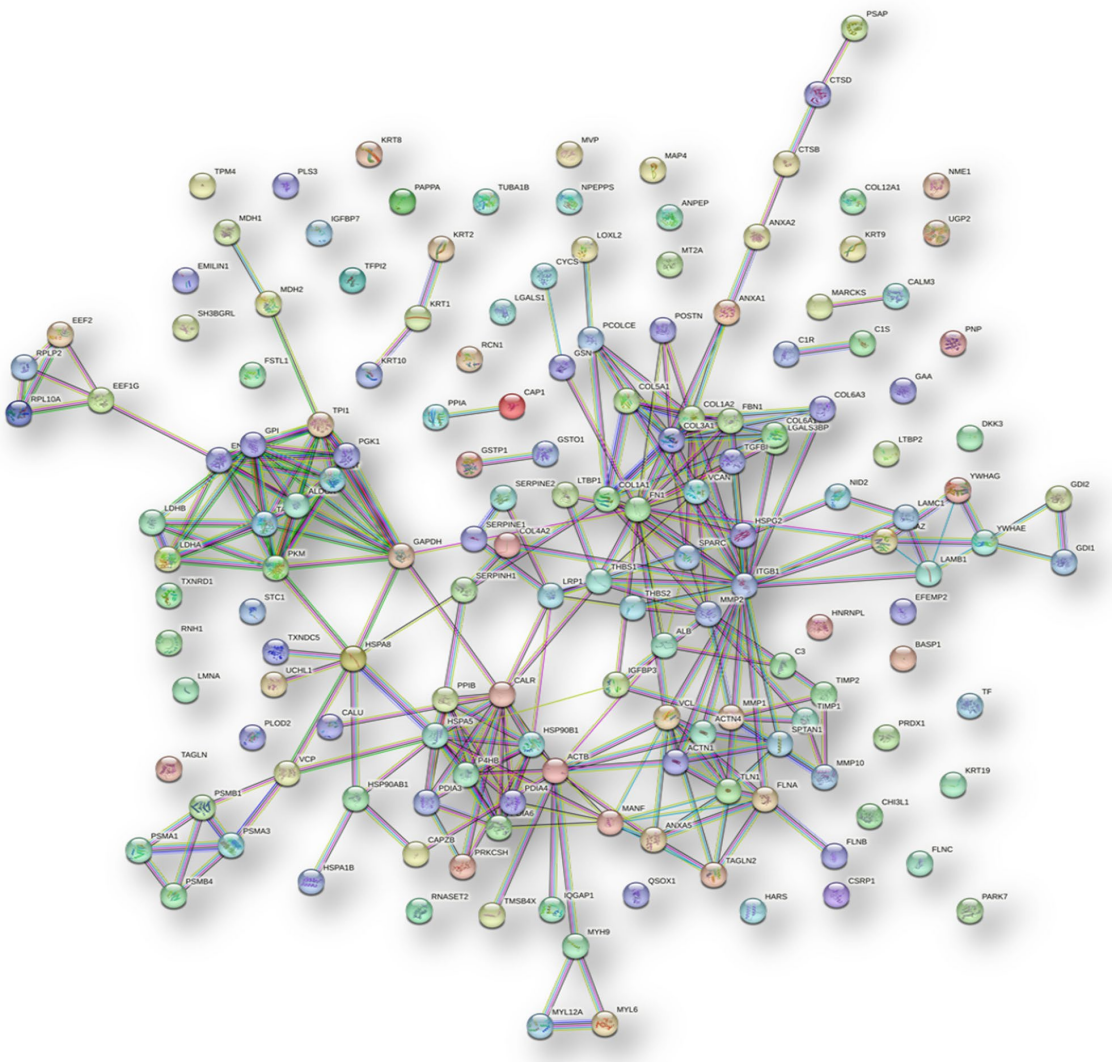
Protein–protein functional interaction network of the 157 proteins commonly identified in hAMSCs secretome (STRING tool analysis, highest confidence)

We finally investigated the predicted network of functional interactions between the 157 proteins common to all hAMSCs secretome pools using the STRING tool (https://string-db.org, accessed on august 23, 2022) (Fig. 4). The network highlights functional and physical associations between the majority of the proteins and revealed 41 clusters significantly enriched, with the top ten listed in Table 3. It is noteworthy that some of these clusters are related to molecular processes involved in collagen formation and synthesis. Additionally, disease-gene associations inside the network revealed 21 significantly enriched diseases, that notably include different bone and cartilage related disorders (Table 4).

**Table 3:** Top ten clusters significantly enriched in the 157 proteins that were repeatedly identified in hAMSC-CM (Fig. 4)

#term	Term description	Observed gene count	Background gene count	Strength	FDR
CL:16,514	Collagen type I trimer, and lateral cystocele	5	5	2.1	1.28e-06
CL:17,150	EF-hand, Ca insensitive, and Vinculin	4	5	2.0	5.96e-05
CL:3175	Sequestering of calcium ion, and disulfide isomerase	4	5	2.0	5.96e-05
CL:16,512	Fibrillar collagen, C-terminal, and Lateral cystocele	6	12	1.79	7.21e-07
CL:16,612	Mixed, incl. g2 nidogen domain and fibulin, and collagen type xii trimer	3	6	1.79	0.0041
CL:17,130	Mixed, incl. profilin binding, and ef-hand, ca insensitive	7	17	1.71	1.31e-07
CL:11,557	Fructose 1,6-bisphosphate metabolic process, and xylulose biosynthetic process	4	10	1.7	0.00041
CL:11,589	Enolase, conserved site, and Phosphoglycerate mutase 1	3	8	1.67	0.0075
CL:16,595	Dissolution of Fibrin Clot, and positive regulation of sterol import	3	8	1.67	0.0075
CL:16,571	Mixed, incl. dissolution of fibrin clot, and negative regulation of metallopeptidase activity	7	20	1.64	2.98e-07

**Table 4:** Disease-gene associations significantly enriched in the proteins functional network (Fig. 4) of the 157 hAMSC secretome pools 1–4 common proteins

#term ID	Term description	Observed gene count	Background gene count	Strength	FDR	Matching proteins in the network (gene name)
DOID:7	Disease of anatomical entity	72	4452	0.3	8.22E-07	VCL,LGALS1,MYH9,MMP2,LAMB1,SERPINE1,COL1A1,GAPDH,TPI1,SPARC,CTSD,C3,KRT9,KRT1,ACTN4,CHI3L1,LAMC1,LTBP2,YWHAE,VCAN,KRT10,PLOD2,UCHL1,CALM3,COL6A3,ALB,COL1A2,PPIB,COL3A1,YWHAG,EEF2,EFEMP2,KRT2,CALR,COL12A1,FBN1,MDH2,FLNC,P4HB,PAPPA,TPM4,CTSB,FN1,ACTB,VCP,COL4A2,PNP,KRT19,COL6A1,THBS2,TAGLN2,LMNA,FLNA,COL5A1,SPTAN1,PGK1,HSPG2,HSPA1B,PSAP,ALDOA,YWHAZ,C1S,GPI,TGFBI,PARK7,FLNB,HARS,SERPINH1,MDH1,C1R,KRT8,PRKCSH
DOID:13,359	Ehlers-Danlos syndrome	7	23	1.58	6.75E-06	COL1A1,COL1A2,COL3A1,COL12A1,COL5A1,C1S,C1R
DOID:65	Connective tissue disease	24	715	0.62	6.78E-06	MMP2,COL1A1,SPARC,PLOD2,COL6A3,COL1A2,PPIB,COL3A1,EFEMP2,COL12A1,FBN1,P4HB,VCP,COL6A1,THBS2,LMNA,FLNA,COL5A1,HSPG2,HSPA1B,C1S,FLNB,SERPINH1,C1R
DOID:12,347	Osteogenesis imperfecta	7	30	1.46	1.30E-05	COL1A1,SPARC,PLOD2,COL1A2,PPIB,P4HB,SERPINH1
DOID:4	Disease	81	5921	0.23	1.69E-05	VCL,LGALS1,MYH9,PSMA3,MMP2,LAMB1,SERPINE1,COL1A1,GAPDH,TPI1,SPARC,CTSD,C3,KRT9,KRT1,ACTN4,CHI3L1,LAMC1,LTBP2,YWHAE,VCAN,KRT10,PLOD2,UCHL1,CALM3,COL6A3,ALB,COL1A2,PPIB,COL3A1,GAA,YWHAG,EEF2,EFEMP2,KRT2,CALR,COL12A1,FBN1,MDH2,FLNC,P4HB,PAPPA,UGP2,TPM4,CTSB,FN1,ACTB,VCP,COL4A2,PNP,KRT19,COL6A1,THBS2,TAGLN2,LMNA,FLNA,COL5A1,SPTAN1,PGK1,GSN,HSPG2,HSPA1B,IGFBP3,PSAP,ALDOA,YWHAZ,C1S,TKT,GDI1,GPI,TGFBI,PARK7,FLNB,HARS,SERPINH1,MDH1,CALU,C1R,LDHA,KRT8,PRKCSH
DOID:2256	Osteochondrodysplasia	10	108	1.06	2.23E-05	COL1A1,SPARC,PLOD2,COL1A2,PPIB,FBN1,P4HB,HSPG2,FLNB,SERPINH1
DOID:17	Musculoskeletal system disease	27	1074	0.5	8.88E-05	MMP2,COL1A1,SPARC,PLOD2,COL6A3,COL1A2,PPIB,COL3A1,EFEMP2,COL12A1,FBN1,FLNC,P4HB,ACTB,VCP,COL6A1,THBS2,LMNA,FLNA,COL5A1,HSPG2,HSPA1B,C1S,FLNB,HARS,SERPINH1,C1R
DOID:9120	Amyloidosis	8	70	1.15	9.57E-05	C3,KRT1,ALB,FN1,ACTB,GSN,HSPG2,TGFBI
DOID:4603	Epidermolytic hyperkeratosis	4	4	2.1	0.00012	KRT9,KRT1,KRT10,KRT2
DOID:0050736	Autosomal dominant disease	27	1163	0.46	0.0003	MYH9,COL1A1,C3,KRT1,ACTN4,YWHAE,KRT10,CALM3,COL6A3,COL1A2,COL3A1,EEF2,KRT2,CALR,FBN1,FLNC,UGP2,VCP,COL4A2,COL6A1,LMNA,FLNA,GSN,HSPG2,TGFBI,FLNB,PRKCSH
DOID:0080006	Bone development disease	11	220	0.79	0.00091	COL1A1,SPARC,PLOD2,COL1A2,PPIB,FBN1,P4HB,FLNA,HSPG2,FLNB,SERPINH1
DOID:0080001	Bone disease	16	523	0.58	0.0022	MMP2,COL1A1,SPARC,PLOD2,COL6A3,COL1A2,PPIB,FBN1,P4HB,VCP,COL6A1,THBS2,FLNA,HSPG2,FLNB,SERPINH1
DOID:0060877	Bullous congenital ichthyosiform erythroderma	3	3	2.1	0.003	KRT1,KRT10,KRT2
DOID:0060158	Acquired metabolic disease	12	320	0.67	0.0042	TPI1,C3,KRT1,ALB,FN1,ACTB,COL6A1,PGK1,GSN,HSPG2,TKT,TGFBI
DOID:14,330	Parkinsons disease	5	51	1.09	0.0212	YWHAE,UCHL1,YWHAG,YWHAZ,PARK7
DOID:174	Acanthoma	3	8	1.67	0.0212	KRT1,KRT10,KRT2
DOID:863	Nervous system disease	34	2132	0.3	0.0212	MYH9,LAMB1,COL1A1,GAPDH,CTSD,C3,LAMC1,LTBP2,YWHAE,VCAN,UCHL1,COL6A3,ALB,YWHAG,EEF2,FBN1,MDH2,CTSB,FN1,ACTB,VCP,COL4A2,LMNA,FLNA,COL5A1,SPTAN1,HSPG2,PSAP,YWHAZ,TGFBI,PARK7,HARS,MDH1,KRT8
DOID:0050739	Autosomal genetic disease	35	2323	0.27	0.0378	MYH9,PSMA3,COL1A1,C3,KRT1,ACTN4,YWHAE,KRT10,CALM3,COL6A3,COL1A2,PPIB,COL3A1,EEF2,EFEMP2,KRT2,CALR,FBN1,FLNC,UGP2,VCP,COL4A2,PNP,COL6A1,LMNA,FLNA,GSN,HSPG2,IGFBP3,PSAP,TGFBI,PARK7,FLNB,HARS,PRKCSH
DOID:0050557	Congenital muscular dystrophy	4	34	1.17	0.0459	COL6A3,COL12A1,COL6A1,LMNA
DOID:630	Genetic disease	41	2962	0.24	0.0488	MYH9,PSMA3,COL1A1,GAPDH,CTSD,C3,KRT1,ACTN4,LTBP2,YWHAE,VCAN,KRT10,CALM3,COL6A3,COL1A2,PPIB,COL3A1,EEF2,EFEMP2,KRT2,CALR,FBN1,FLNC,PAPPA,UGP2,VCP,COL4A2,PNP,COL6A1,LMNA,FLNA,PGK1,GSN,HSPG2,IGFBP3,PSAP,TGFBI,PARK7,FLNB,HARS,PRKCSH
DOID:90	Degenerative disc disease	3	13	1.46	0.0488	MMP2, COL1A1,THBS2

Next, we investigated the classification/prediction of secreted proteins among the 157 commonly-identified proteins, using the Human Protein Atlas (HPA) Subcellular Section—Secreted proteins (2793 genes) as a reference (http://www.proteinatlas.org, accessed on October 13, 2022). Seventy-nine proteins (50.3%) were classified/predicted as secreted (Additional file 3*:* Table S3A). Among these, 27 were subclassified as “proteins secreted to extracellular matrix”, 27 as “proteins secreted to blood”, 20 as “intracellular and membrane proteins”, 3 as “proteins secreted in other tissues”, and 2 as “proteins secreted in female reproductive system” (data in Additional file 3: Table S3B).

**Fig. 5:**
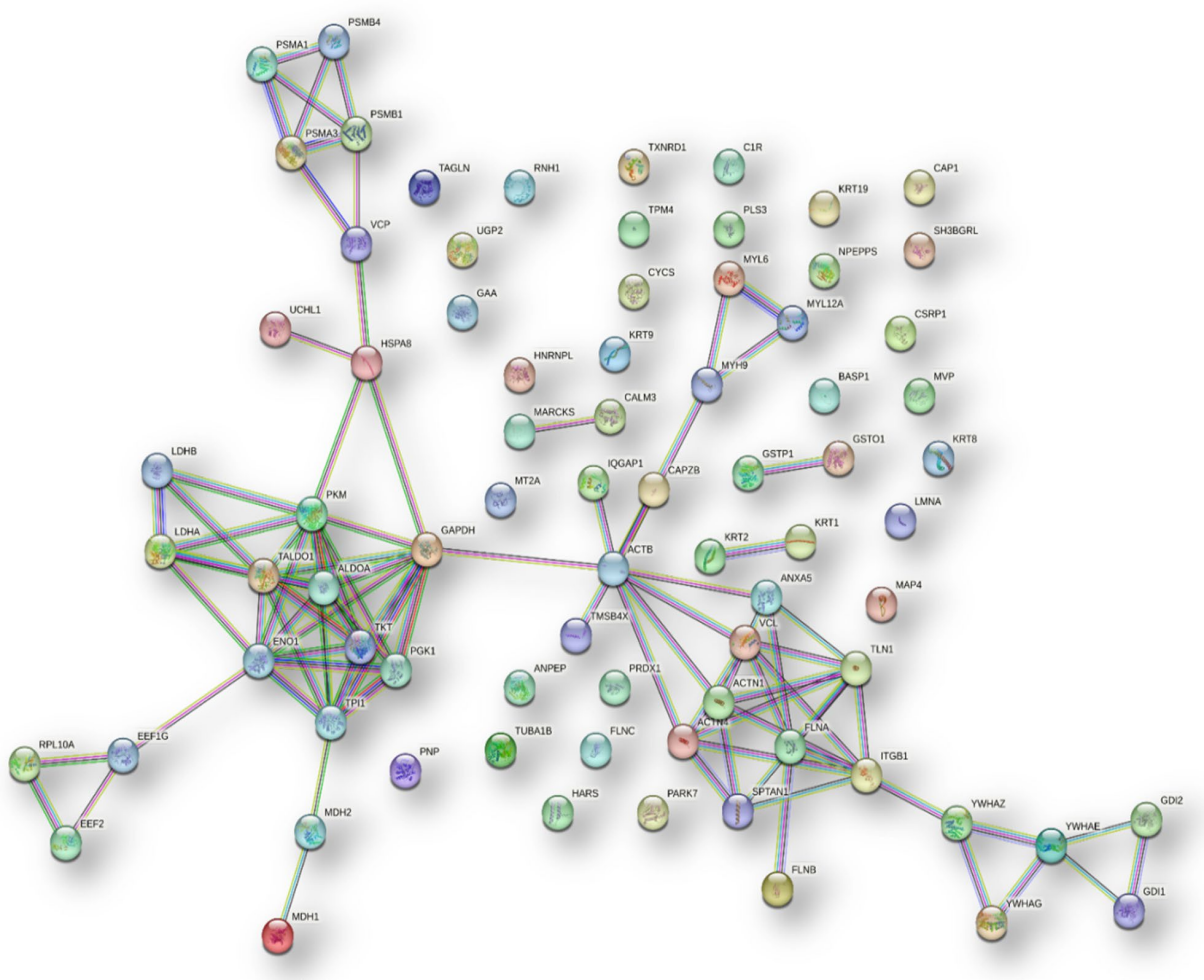
Protein–protein functional interaction network of the 78 out of the 157 commonly identified proteins in hAMSCs secretome which were not classified as secreted proteins (STRING tool analysis, highest confidence)

The remaining 78 proteins, which were not classified/predicted as secreted proteins, were further investigated for their potential functional relationships and for enriched clusters of interactions using STRING (https://string-db.org, accessed on August 23, 2022). Notably, clusters related to carbon metabolism were among the top ten most significantly enriched (Fig. 5, Table 5).

**Table 5:** List of the top ten clusters significantly enriched among the 78 proteins (Fig. 5) not classified as secreted proteins

#term ID	Term description	Observed gene count	Background gene count	Strength	FDR	Matching proteins in your network (gene name)
CL:11,548	Carbon metabolism, and Starch and sucrose metabolism	13	124	1.42	3.52E-11	TPI1,ENO1,GAA,PKM,TALDO1,MDH2,UGP2,PGK1,ALDOA,LDHB,TKT,MDH1,LDHA
CL:17,048	RHO GTPases Activate WASPs and WAVEs, and actin filament organization	12	105	1.46	7.94E-11	VCL,ACTN4,IQGAP1,ANXA5,TLN1,PLS3,ACTB,FLNA,CAP1,TMSB4X, ACTN1,FLNB
CL:11,549	Carbon metabolism, and Pyruvate metabolism	11	96	1.46	3.22E-10	TPI1,ENO1,PKM,TALDO1,MDH2,PGK1,ALDOA,LDHB,TKT,MDH1,LDHA
CL:11,551	Pentose phosphate pathway, and Glycolysis	9	55	1.61	2.29E-09	TPI1,ENO1,PKM,TALDO1,PGK1,ALDOA,LDHB,TKT,LDHA
CL:17,127	Mixed, incl. profilin binding, and profilin	7	33	1.73	9.01E-08	VCL,ACTN4,IQGAP1,TLN1,ACTB,TMSB4X, ACTN1
CL:17,130	Mixed, incl. profilin binding, and ef-hand, ca insensitive	6	17	1.95	1.27E-07	VCL,ACTN4,TLN1,ACTB,TMSB4X,ACTN1
CL:17,049	RHO GTPases Activate WASPs and WAVEs, and actin filament organization	9	96	1.37	1.28E-07	VCL,ACTN4,IQGAP1,TLN1,PLS3,ACTB,CAP1,TMSB4X,ACTN1
CL:11,554	Glycolysis, and Fructose-1,6-bisphosphatase	6	31	1.69	2.20E-06	TPI1,ENO1,TALDO1,PGK1,ALDOA,TKT
CL:17,150	EF-hand, Ca insensitive, and Vinculin	4	5	2.3	8.11E-06	VCL,ACTN4,TLN1, ACTN1
CL:17,373	Muscle protein, and sarcomere organization	6	68	1.35	0.00012	MYH9,MYL12A,FLNC,TPM4,TAGLN,MYL6

**Fig. 6:**
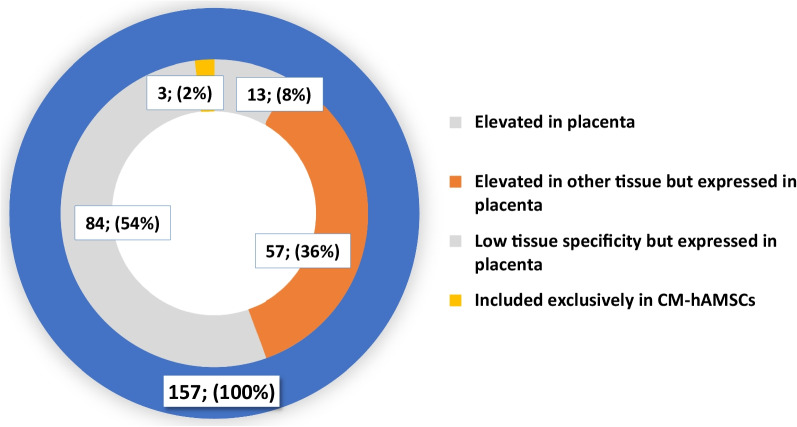
Distribution of the 157 commonly identified proteins in all hAMSCs-CM based on placenta gene classification as i) elevated expression in placenta; ii) elevated expression in other tissues but expressed in placenta; iii) low tissue specificity but expression in placenta and iv) proteins identified in hAMSCs not classified as placenta proteins.The number of proteins and the relative percent value (versus total 157) are shown

Transcriptomics data revealed that 65% (*n* = 13,060) of all human proteins (*n* = 20,090) are expressed in placenta [33], and 286 of these have an elevated expression when compared to other tissues. It was therefore interesting to determine how many of the 157 proteins that characterized the hAMSCs secretome were classified as placental proteins. To do this, we referred to the HPA placenta proteome database (https://www.proteinatlas.org/humanproteome/tissue/placenta, accessed on October 13, 2022). The donut chart in Fig. 6 illustrates the distribution of the 157 proteins (outer blue ring) in relation to their classification as “elevated expression in placenta”, or “elevated in other tissues but expressed in placenta”, or “low tissue specificity but expressed in placenta”. The results demonstrate that 13 elements (8%) were classified to have an elevated expression in placenta, 57 (36%) with elevated expression in other tissues but expressed in placenta, and 84 elements (54%) expressed in the placenta but with low tissue specificity (data analysis in Additional file 4: Table S4). Only 3 out of the 157 proteins, namely keratins type I cytoskeletal 9, keratin type II cytoskeletal 2 epidermal, and complement C1r subcomponent, were not classified as genes expressed in placenta. Complement C1r subcomponent is involved in the assembling of complement C1, the first component of the classical pathway of the complement system of the innate immune system. On the other hand, both keratins were identified in the control medium (DMEMF12) and therefore they can be excluded from the components of the hAMSCs secretome.

It is noteworthy that the 13 genes with elevated expression in placenta were all classified as secretome proteins in the HPA database (Venn diagram grouping analysis in Additional file 4: Table S4). These genes include tissue factor pathway inhibitor 2, pappalysin-1, glia-derived nexin, collagen alpha -1(III) chain, collagen alpha-2(IV) chain, fibrillin-1, fibronectin, insulin-like growth factor-binding protein 3, laminin subunit gamma-1, nidogen-2, plasminogen activator inhibitor 1, SPARC, and transforming growth factor-beta-induced protein ig-h3. SPARC is a 32 kDa calcium-binding matricellular multifunctional glycoprotein [34], whose expression is closely associated with that of fibrillar collagens, such as type I collagen. This protein acts more as a regulator of cellular behavior rather than as a structural component of the ECM, and is involved in tissue remodeling, repair, development, and cellular turnover [35].

### Pathway enrichment analysis in the secretome of hAMSCs cells

**Fig. 7:**
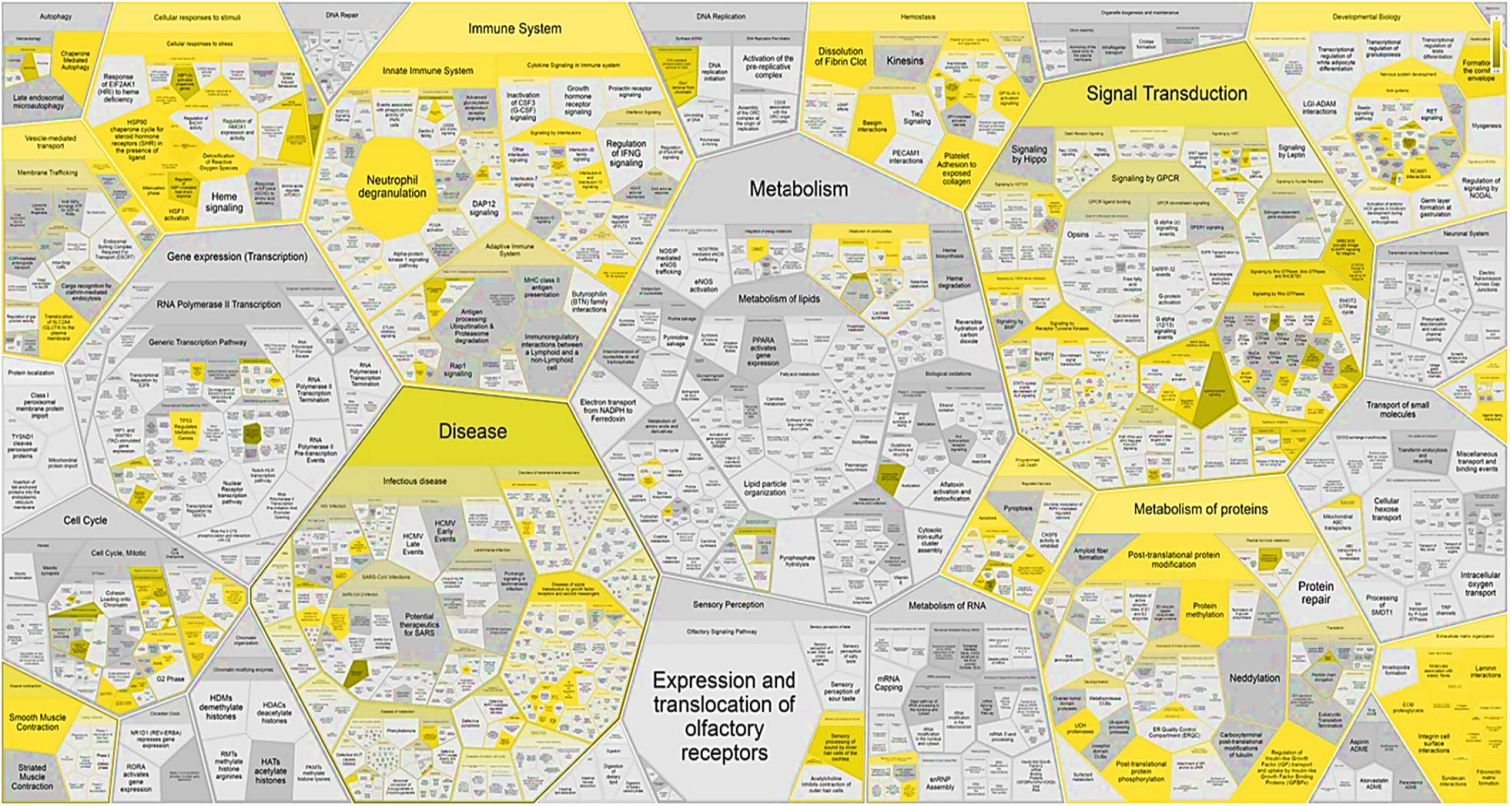
Voronoi diagram representation of the hierarchical pathways overrepresentations analysis by Reactome of the 157 commonly-identified protein elements of hAMSC-CM s

The molecular pathways over-representation analysis of the 157 commonly-identified proteins in the hAMSCs secretome was performed using Reactome (https://reactome.org, accessed on September 21, 2022). Figure 7 shows the Voronoi diagram representation of the results obtained. The diagram highlights the over-represented hierarchical pathways, including the Immune system, Signal transduction, Gene expression (Transcription), Hemostasis, Developmental biology, DNA repair, Disease, Extracellular matrix organization, Cellular responses to stimuli, and Nephrin family interactions. Some of these pathways are extremely important in regenerative processes, thus we further investigated their protein elements.

The Immune system pathway plays a critical role in directing tissue repair and regeneration outcomes [36]. Sixty-three out of the 157 commonly-identified proteins were involved in this pathway (list in Table 6). The disease gene annotation performed using the STRING tool showed an enrichment of genes associated with various conditions, including Ehlers-Danlos syndrome, Amyloidosis, Connective tissue disease, Disease of anatomical entity, Autosomal dominant disease, Osteogenesis imperfecta type 1, Osteogenesis imperfecta type 4, and Otopalatodigital syndrome type.

**Table 6:** List of the 63 proteins involved in the Immune system pathway

Protein ID	Protein name	Protein ID	Protein name
P02795	Metallothionein-2	P14618	Pyruvate kinase PKM
P62937	Peptidyl-prolyl cis–trans isomerase A	P07602	Prosaposin
P01033	Metalloproteinase inhibitor 1	P14314	Glucosidase 2 subunit beta
P09211	Glutathione S-transferase P	P06744	Glucose-6-phosphate isomerase
P16035	Metalloproteinase inhibitor 2	P04264	Keratin, type II cytoskeletal 1
P20618	Proteasome subunit beta type-1	P0DMV8	Heat shock 70 kDa protein 1A
P78417	Glutathione S-transferase omega-1	P11142	Heat shock cognate 71 kDa protein
P63104	14–3-3 protein zeta/delta	P11021	Endoplasmic reticulum chaperone BiP
P25788	Proteasome subunit alpha type-3	P08253	72 kDa type IV collagenase
P28070	Proteasome subunit beta type-4	P09871	Complement C1s subcomponent
O00584	Ribonuclease T2	P00736	Complement C1r subcomponent
P25786	Proteasome subunit alpha type-1	O00391	Sulfhydryl oxidase 1
P47756	F-actin-capping protein subunit beta	P08238	Heat shock protein HSP 90-beta
P00491	Purine nucleoside phosphorylase	P06396	Gelsolin
P37837	Transaldolase	P05556	Integrin beta-1
P07858	Cathepsin B	P55072	Transitional endoplasmic reticulum ATPase
P07355	Annexin A2	P14625	Endoplasmin
P04083	Annexin A1	P13639	Elongation factor 2
P04075	Fructose-bisphosphate aldolase A	Q14764	Major vault protein
P60709	Actin, cytoplasmic 1	P55786	Puromycin-sensitive aminopeptidase
P36222	Chitinase-3-like protein 1	P10253	Lysosomal alpha-glucosidase
P07339	Cathepsin D	P15144	Aminopeptidase N (CD13)
Q8NBS9	Thioredoxin domain-containing protein 5	P18206	Vinculin
P27797	Calreticulin	P08123	Collagen alpha-2(I) chain
P68363	Tubulin alpha-1B chain	P02461	Collagen alpha-1(III) chain
P50395	Rab GDP dissociation inhibitor beta	P02452	Collagen alpha-1(I) chain
Q01518	Adenylyl cyclase-associated protein 1	P01024	Complement C3
P03956	Interstitial collagenase	P46940	Ras GTPase-activating-like protein IQGAP1
P30101	Protein disulfide-isomerase A3	P35579	Myosin-9
P07237	Protein disulfide-isomerase	P02751	Fibronectin
P21333	Filamin-A	O75369	Filamin-B
Q13813	Spectrin alpha chain, non-erythrocytic 1		

Another over-represented pathway identified in the hAMSCs secretome is the Hemostasis pathway. This pathway plays a crucial role in the physiological response that ultimately leads to the arrest of bleeding from an injured vessel [37]. A total of 34 proteins resulted involved in this pathway (Table 7) and, among these, STRING analysis revealed 13 significantly enriched clusters. These clusters include “RHO GTPases Activate WASPs and WAVEs, and actin filament organization” (CL:17,048), “Mixed, incl. profilin binding, and ef-hand, ca insensitive”(CL:17,130), “RHO GTPases Activate WASPs and WAVEs, and actin filament organization” (CL:17,049), “Collagen formation, and Defective B3GALTL causes Peters-plus syndrome (PpS)”( CL:16,429), “EF-hand, Ca insensitive, and Vinculin”(CL:17,150), “Collagen formation, and Matrix metalloproteinases”(CL:16,430), “Mixed, incl. dissolution of fibrin clot, and negative regulation of metallopeptidase activity”(CL:16,571), “Collagen type i trimer, and lateral cystocele”(CL:16,514), “Mixed, incl. conjunctivochalasis, and metalloproteinase inhibitor 1”(CL:16,575), “Integrin alpha5-beta1 complex, and integrin alphav-beta6 complex”(CL:16,873), “Profilin conserved site, and Baraitser-Winter syndrome”(CL:17,132), “Mixed, incl. annexin a5, and transgelin-2”(CL:17,241), and “Dissolution of Fibrin Clot, and positive regulation of sterol import”(CL:16,595).

**Table 7:** List of the 34 proteins involved in the Hemostasis pathway

Protein ID	Protein name	Protein ID	Protein name
P62328	Thymosin beta-4	P60709	Actin, cytoplasmic 1
P62937	Peptidyl-prolyl cis–trans isomerase A	P07093	Glia-derived nexin
P55145	Mesencephalic astrocyte-derived neurotrophic factor	P05121	Plasminogen activator inhibitor 1
P37802	Transgelin-2	P68363	Tubulin alpha-1B chain
P01033	Metalloproteinase inhibitor 1	Q01518	Adenylyl cyclase-associated protein 1
P63104	14–3-3 protein zeta/delta	P03956	Interstitial collagenase
P47756	F-actin-capping protein subunit beta	P07602	Prosaposin
P09486	SPARC	Q08380	Galectin-3-binding protein
P08758	Annexin A5	P02768	Albumin
O43852	Calumenin	P11021	Endoplasmic reticulum chaperone BiP
P07355	Annexin A2	P02787	Serotransferrin
P04075	Fructose-bisphosphate aldolase A	O00391	Sulfhydryl oxidase 1
P07996	Thrombospondin-1	P05556	Integrin beta-1
P02452	Collagen alpha-1(I) chain	P12814	Alpha-actinin-1
Q9Y490	Talin-1	O43707	Alpha-actinin-4
P02751	Fibronectin	P18206	Vinculin
P21333	Filamin-A	P08123	Collagen alpha-2(I) chain

The pathway of Developmental biology includes several developmental processes such as the transcriptional regulation of pluripotent stem cells, gastrulation, and the activation of HOX genes during differentiation. A total of 31 proteins resulted to be involved in this important pathway (Table 8) and, among these, STRING analysis revealed16 significantly enriched clusters. The clusters included “Collagen formation, and Matrix metalloproteinases”, “Collagen biosynthesis and modifying enzymes”, “Proteasome”, “Keratin type II head”, “Proteasome subunit, and proteasome regulatory particle”, “Myosin ii complex, and rho gtpases activate rocks”, “Collagen biosynthesis and modifying enzymes”, “Bethlem myopathy, and NCAM1 interactions”, “EF-hand domain, and myosin II filament”, “Keratin”, “Mixed, incl. laminin-10 complex, and sprouting of injured axon”, “Protein complex involved in cell adhesion, and met activates ptk2”, “signalling”, “Fibrillar collagen, C-terminal, and Lateral cystocele”, “Mixed, incl. cystadenoma, and intrahepatic cholangiocarcinoma”, “Proteasome regulatory particle, and proteasome subunit”, and “Mixed, incl. profilin binding, and ef-hand, ca insensitive”. Some of these clusters are shared with the enriched clusters of proteins involved in the Hemostasis pathway.

**Table 8:** List of the 31 proteins involved in the Developmental biology pathway

Protein ID	Protein name
P05387	60S acidic ribosomal protein P2
P60660	Myosin light polypeptide 6
P19105	Myosin regulatory light chain 12A
P62906	60S ribosomal protein L10a
P20618	Proteasome subunit beta type-1
P25788	Proteasome subunit alpha type-3
P28070	Proteasome subunit beta type-4
P25786	Proteasome subunit alpha type-1
P60709	Actin, cytoplasmic 1
P08727	Keratin, type I cytoskeletal 19
P68363	Tubulin alpha-1B chain
Q01518	Adenylyl cyclase-associated protein 1
P05787	Keratin, type II cytoskeletal 8
P13645	Keratin, type I cytoskeletal 10
P35527	Keratin, type I cytoskeletal 9
P35908	Keratin, type II cytoskeletal 2 epidermal
P04264	Keratin, type II cytoskeletal 1
P11142	Heat shock cognate 71 kDa protein
P08253	72 kDa type IV collagenase
P08238	Heat shock protein HSP 90-beta
P05556	Integrin beta-1
P12109	Collagen alpha-1(VI) chain
P02461	Collagen alpha-1(III) chain
P08572	Collagen alpha-2(IV) chain
P11047	Laminin subunit gamma-1
P20908	Collagen alpha-1(V) chain
P07942	Laminin subunit beta-1
P35579	Myosin-9
Q9Y490	Talin-1
Q13813	Spectrin alpha chain, non-erythrocytic 1
P12111	Collagen alpha-3(VI) chain
Q13813	Spectrin alpha chain, non-erythrocytic 1

The pathway of Extracellular matrix organization is involved in the regulation of cell differentiation processes, such as the establishment and maintenance of stem cell niches, branching morphogenesis, angiogenesis, bone remodeling, and wound repair [38]. A total of 37 proteins were found to be involved in this pathway (Table 9) and, among these, STRING analysis revealed the enrichment of clusters mostly related to collagen biosynthesis and degradation (Table 9). Among these, collagen is most abundant fibrous protein in the ECM that constitutes up to 30% of total proteins in multicellular animals. It provides tensile strength and it is associated with elastic fibers composed of elastin and fibrillin microfibrils, which give tissues the ability to recover after stretching. Other ECM proteins, such as fibronectin, laminins, and matricellular proteins, participate as connectors or linking proteins [38], (Table 9).

**Table 9:** List of the 37 proteins involved in the Extracellular matrix organization

Protein ID	Protein name
P01033	Metalloproteinase inhibitor 1
P23284	Peptidyl-prolyl cis–trans isomerase B
P16035	Metalloproteinase inhibitor 2
P09486	SPARC
P07858	Cathepsin B
P07339	Cathepsin D
P05121	Plasminogen activator inhibitor 1
P50454	Serpin H1
Q15113	Procollagen C-endopeptidase enhancer 1
O95967	EGF-containing fibulin-like extracellular matrix protein 2
P03956	Interstitial collagenase
P09238	Stromelysin-2
P07237	Protein disulfide-isomerase
P08253	72 kDa type IV collagenase
O00469	Procollagen-lysine,2-oxoglutarate 5-dioxygenase 2
Q9Y4K0	Lysyl oxidase homolog 2
P05556	Integrin beta-1
P12814	Alpha-actinin-1
Q9Y6C2	EMILIN-1
P12109	Collagen alpha-1(VI) chain
P08123	Collagen alpha-2(I) chain
P07996	Thrombospondin-1
P02461	Collagen alpha-1(III) chain
P02452	Collagen alpha-1(I) chain
Q14112	Nidogen-2
P08572	Collagen alpha-2(IV) chain
P11047	Laminin subunit gamma-1
P20908	Collagen alpha-1(V) chain
Q14766	Latent-transforming growth factor beta-binding protein 1
Q14767	Latent-transforming growth factor beta-binding protein 2
P07942	Laminin subunit beta-1
P02751	Fibronectin
P35555	Fibrillin-1
Q99715	Collagen alpha-1(XII) chain
P12111	Collagen alpha-3(VI) chain
P13611	Versican core protein
P98160	Basement membrane-specific heparan sulfate proteoglycan core protein

The pathway of Cellular responses to stimuli is essential for normal development, maintenance of homeostasis in mature tissues, and effective defensive responses to potentially noxious agents [39]. A total of 30 proteins were found to be involved in this pathway (Table 10) and, among these, STRING analysis revealed 8 significantly enriched clusters. These clusters include “Photodynamic therapy-induced unfolded protein response, and protein disulfide isomerase activity”, “Photodynamic therapy-induced unfolded protein response, and Inhibition of PKR”, “Protein processing in endoplasmic reticulum, and Insertion of tail-anchored proteins into the endoplasmic reticulum membrane”, “Sequestering of calcium ion, and disulfide isomerase”, “Proteasome”, “Proteasome subunit, and proteasome regulatory particle”, “Chaperone complex, and chaperone cofactor-dependent protein refolding”, and “Fructose 1,6-bisphosphate metabolic process, and xylulose biosynthetic process”.

**Table 10:** List of the 30 proteins involved in the pathway of Cellular responses to stimuli

Protein ID	Protein name	Protein ID	Protein name
P05387	60S acidic ribosomal protein P2	Q15084	Protein disulfide-isomerase A6
P99999	Cytochrome c	P27797	Calreticulin
P02795	Metallothionein-2	P68363	Tubulin alpha-1B chain
P21291	Cysteine and glycine-rich protein 1	P07237	Protein disulfide-isomerase
Q06830	Peroxiredoxin-1	P29401	Transketolase
P09211	Glutathione S-transferase P	P02768	Albumin
P62906	60S ribosomal protein L10a	P0DMV8	Heat shock 70 kDa protein 1A
P20618	Proteasome subunit beta type-1	P11142	Heat shock cognate 71 kDa protein
P25788	Proteasome subunit alpha type-3	Q16881	Thioredoxin reductase 1, cytoplasmic
Q16270	Insulin-like growth factor-binding protein 7	P11021	Endoplasmic reticulum chaperone BiP
P62258	14–3-3 protein epsilon	P02545	Prelamin-A/C
P28070	Proteasome subunit beta type-4	P08238	Heat shock protein HSP 90-beta
P25786	Proteasome subunit alpha type-1	P55072	Transitional endoplasmic reticulum ATPase
P47756	F-actin-capping protein subunit beta	P14625	Endoplasmin
P37837	Transaldolase	Q9Y490	Talin-1

### Evaluation of the most abundant proteins in the hAMSCs secretome

**Fig. 8:**
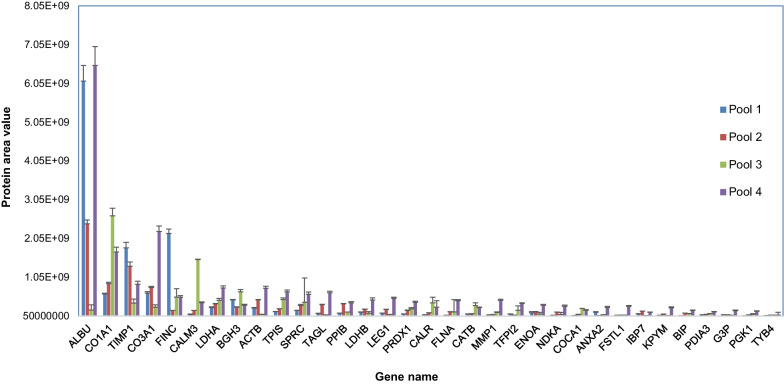
Histogram of the relative quantitation of the most abundant proteins identified inside the group of 157 commonly-identified proteins in the hAMSCs secretome. The average protein area values and the stabndard deviation as resulting from analytical triplicate analysis are reported on the y-axis

Finally, we identified the most abundant proteins among the 157 commonly-identified proteins in the hAMSCs secretome. We thus applied a filter to the protein area data obtained from the Proteome Discoverer analysis of the LC–MS raw files, specifically considering protein area values ≥ 5 × 10^7^. This resulted in a total of 33 proteins that showed protein area values in the range 5 × 10^7^–1 × 10^9^ (Additional file 5*:* Table S5A and B). These proteins collectively constitute the most abundant proteins of the hAMSCs secretome. Figure 8 illustrates the label-free relative quantitation graph for these proteins across the four hAMSCs secretome pools. This quantitation is based on the average protein area values obtained from LC–MS analysis performed in triplicate. These data provide valuable insights into the proteins that significantly contribute to the overall protein profile of the hAMSC secretome.

**Fig. 9:**
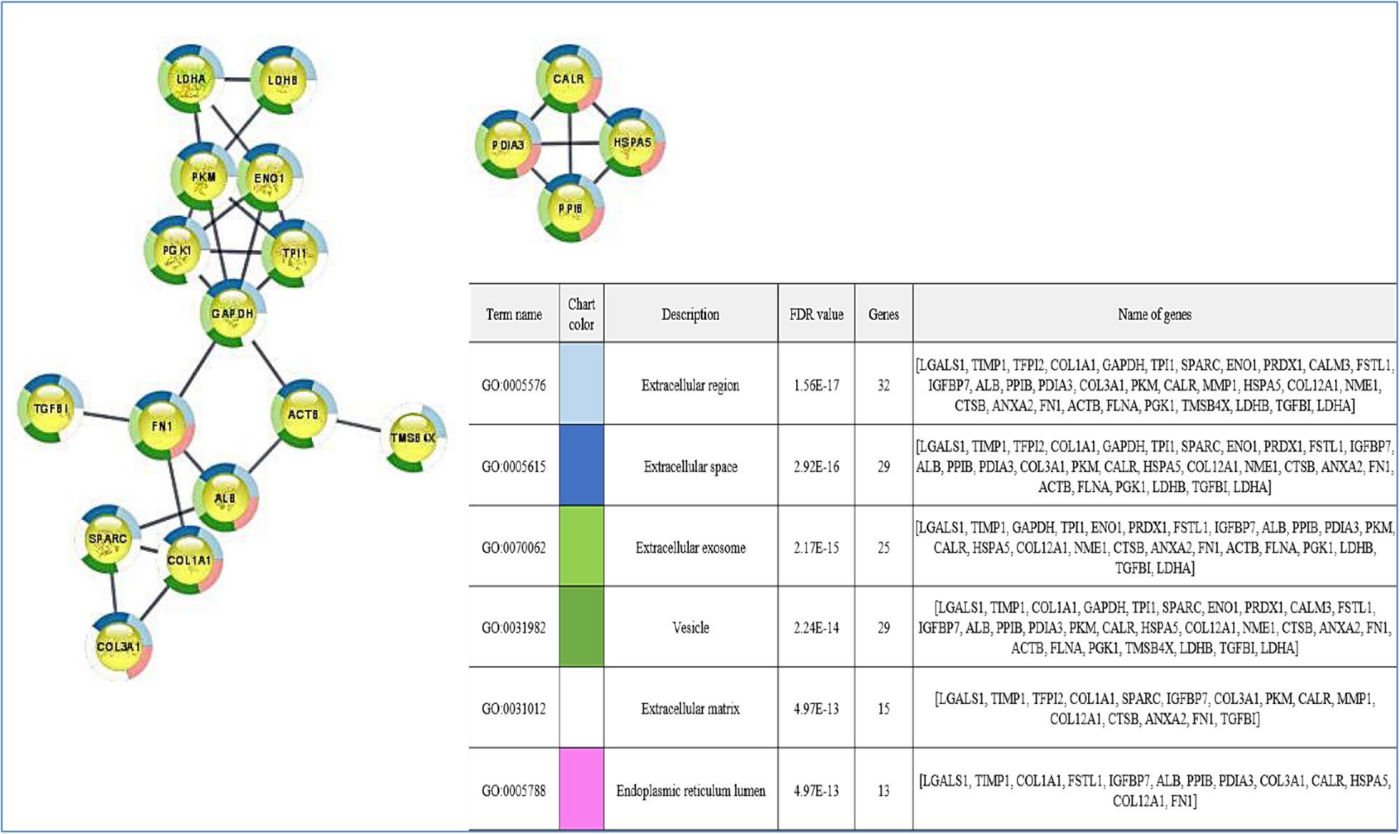
Functional interaction network and biological pathways of the most abundant proteins of the hAMSC secretome

This group of abundant proteins was further analysed using STRING and Cytoscape bioinformatic tools (https://cytoscape.org) to discover specific functional relationships. Figure 9 illustrates the Cytoscape-STRING network, which represents interactions among the 33 most abundant proteins in the hAMSCs secretome. Additionally, it highlights the top 6 enriched terms, which are annotated in different colours at the nodes. The results demonstrate that each protein appears to be involved in multiple categories. Interestingly, a large number of the abundant proteins are classified as components of the Extracellular region, Extracellular space, matrix, and exosomes and vescicle categories.

## Discussion

To enhance our understanding of the therapeutic mechanisms of mesenchymal stromal cells (MSC) in regenerative medicine and immune-related disorders, we conducted an in-depth analysis of the proteins secreted by human amniotic membrane-derived MSCs (hAMSCs). In the hAMSCs secretome, we identified 157 highly abundant proteins, 79 of which are reported as secreted in the Human Protein Atlas (HPA) database, and the remaining 78 have not been previously categorized as secreted proteins. Reactome analysis unveiled several pathways prominently represented in the hAMSCs secretome, including Immune system, Signal transduction, Gene expression (transcription), Hemostasis, Developmental biology, DNA repair, Disease, Extracellular matrix organization, Cellular responses to stimuli, and Nephrin family interactions. Some of these pathways, such as Extracellular matrix organization, Hemostasis, and Immune system, directly relate to processes like tissue remodeling, particularly evident in wound healing [40–42]. The significant presence of elements associated with the Developmental biology pathway may stem from the fetal origin of hAMSCs [43, 44].

Among the proteins in the Immune system cluster, we found those with immunoregulatory action such as metallothionein-2 (MT2), glutathione S-transferase Omega 1, elongation factor 2 (eEF-2 K), various immunoproteasome-related proteins, and complement proteins.

Metallothioneins (MT) have been reported to play an immunoregulatory role in autoimmune diseases, infections, and inflammatory bowel diseases [45, 46]. Among the MT, MT2 showed better outcomes in a mouse model of autoimmune encephalomyelitis, preserving myelin and reducing neuroinflammation compared to MT1 [47]. In an acute lung injury model, MT knockout (-/-) mice displayed increased edema, proinflammatory molecules, and NF-κB nuclear localization compared to wildtype mice [48]. MT also protected against inflammatory organ damage in a study with MT knockout mice, reducing prothrombin, C-reactive protein, and fibrinogen production after LPS exposure [49]. Furthermore, in vitro studies evidenced that MT can inhibit the proliferation of cytotoxic T lymphocytes, as also previously reported for macrophages and lymphocytes [50, 51], possibly by interfering with cell–cell interactions, resulting in immature T lymphocytes and reduced differentiation to effector CTLs [52]. eEF-2 K is another protein with immune-regulatory action identified in the hAMSCs secretome. CD8 lymphocytes from eEF-2 K KO mice show increased proliferation but reduced post-activation survival, likely due to premature induction of senescence via hyperactivation of the Akt-mTOR-S6K pathway [53]. In addition, several proteins in hAMSCs secretome identified in the Immune cluster have been shown to be crucial for the activation of immune responses. For example, some immunoproteasome-related proteins (Proteasome subunit beta type-1, Proteasome subunit alpha type-3, Proteasome subunit beta type-4, Proteasome subunit alpha type-1) which have been reported to be crucial for inflammatory T helper lymphocyte differentiation and implicated in autoimmune disease pathogenesis [54, 55]. The enzyme glutathione S-transferase omega 1 (GSTO1-1), which has been reported to play a pro-inflammatory role in response to LPS [56]. Furthermore, knockdown of GSTO1-1 in macrophage-like cells was shown to block NADPH oxidase 1 expression and ROS generation after LPS stimulation. Paradoxically, GSTO1-1-deficient mice exhibited a more severe inflammatory response and increased bacterial escape in a model of inflammatory bowel disease [57]. Other proteins identified in the hAMSCs secretome that were reported to be crucial for immune responses are complement protein C3 and their proteolytic products C3a, which has chemotactic properties, and C3b which stimulates the innate immune response by opsonizing pathogens. C3 combined with collagen type 1 has been shown to boost inflammation, collagen deposition, and wound healing [58]. Among the other proteins represented in the Immune cluster, are tissue inhibitor of metalloproteinases 1 and 2 (TIMP1 and TIMP2), which are reported to exert an immunoregulatory action and also play a major role in matrix remodeling. During wound healing, keratinocytes produce TIMP1 and TIMP2 to promote tissue remodeling and homeostasis, but dysregulated expression can lead to fibrosis [59]. Increased TIMP1 levels, for example, enhance wound healing and have an antiapoptotic effect in diabetic patients [60]. On the other hand, TIMP levels can sometimes decrease in diseased tendons [61], and TIMP1 can inhibit ECM degradation through MMP2 [62]. Interestingly, a key protein known to be involved in the immunomodulatory actions of MSCs, namely indoleamine 2,3 dioxygenase (IDO), which is known to inhibit lymphocyte responses, was not detected in our analysis. This absence of detection could be attributed to the stringent limitations of the analytical method we applied, which exceeded the protein's limit of detection.

Other highly represented proteins in the hAMSC secretome were clustered in the Hemostasis pathway and have also been reported to possess immunomodulatory properties. These proteins include transgelin-2, thymosin beta 4, thrombospondin 1, talin-1, filamin A (FlnA), and Galectin 3 binding protein. Transgelin-2 stabilizes cytoskeletal actin, facilitating T-cell synapse interactions with antigen-presenting cells [71]. Thrombospondin-1 (TSP-1) is transiently released in large quantities by neutrophils during the initial stages of acute inflammation and can exert a strong chemotactic action [63]. Its action is mainly carried out by inducing a strong inflammatory action that accelerates the repair process by facilitating the phagocytosis of damaged cells and the generation of T regulatory cells [64, 65]. Thus, TSP-1 could represent a compensatory mechanism for controlling the immune response and protecting tissues from excessive damage. Talin-1 has been shown to maintain T regulatory cell homeostasis since its absence in CD4 lymphocytes led to spontaneous activation due to decreased T regulatory cell levels [66]. FlnA, has been shown to affect T lymphocyte adhesion and infiltration into tissues, indirectly impacting their function [67]. Galectin 3 plays a multifaceted role in regulating the inflammatory response by influencing macrophage polarization [68], angiogenesis [69], and fibroblast-to-myofibroblast conversion [70], making it pivotal in wound healing processes [71]. Thymosin beta 4 participates in recruiting stem and progenitor cells, promoting cardiac repair after myocardial infarction [72, 73]. It also interferes with TNF-α-mediated NF-κB activation and IL-8 gene transcription, contributing to immunomodulation [74]. Other proteins identified in the hemostasis cluster, but without immunomodulatory actions, are mesencephalic astrocyte-derived neurotrophic factor (MANF) and secreted protein acidic and rich in cysteine (SPARC). MANF is a neurotrophic factor reported to exert protective actions in various central nervous system diseases [75]. For example, pretreatment with MANF before middle cerebral artery occlusion in rats has been shown to improve locomotor abilities and reduced neurological deficits [76]. MANF has also shown benefit in Parkinson's and Alzheimer's disease by protecting dopaminergic neurons and reducing intracellular α-synuclein aggregates in Parkinson's disease [77] and mitigating Aβ1-42-induced neuronal cell death in Alzheimer's disease [78]. SPARC, a matrix glycoprotein, with diverse functions which is highly expressed during tissue damage, inflammation, and in tumors [79], and was shown to inhibit angiogenesis by interfering with the binding of pro-angiogenic factors such as VEGF, PDGF, and bFGF to their receptors, thus countering blood vessel formation [80]. In regenerative medicine, exogenous SPARC was shown to accelerate the proliferation of limbal epithelial stem cells and promote their migration, expediting corneal wound healing in in vivo corneal damage models [81].

Furthermore, proteins found in the Extracellular matrix organization cluster, such as laminin, collagen, and fibronectin, play crucial roles in regeneration processes. Laminin can facilitate cell interactions with other extracellular matrix components, such as collagen and heparin sulfate [82]. It can serve as a substrate for the migration of epithelial keratinocytes during re-epithelialization [83] and contribute to the formation and maturation of blood vessels, particularly in processes like neoangiogenesis [84]. Collagen, a fundamental extracellular matrix protein, is essential for wound healing [85]. It undergoes accelerated turnover during skin remodeling and healing. However, alterations in collagen can lead to functional changes in repairing tissue, potentially resulting in fibrosis [86]. Fibronectin can play a significant role in mediating hemostasis and the migration and recruitment of cell progenitors during wound healing processes [87–89].

## Conclusions

In conclusion, our analysis has revealed specific proteins within the hAMSCs secretome that fall into clusters with significantly enriched interactions. Nevertheless, it's important to acknowledge the existence of many other proteins whose functional roles in regenerative medicine remain undefined. What emerges from our study is the remarkable complexity of the hAMSCs secretome, whereby we observe proteins with distinct but also paradoxical actions. Yet, it's worth noting that this coexistence of proteins with seemingly contradictory roles may be essential to maintain a dynamic balance between inflammatory and immunoregulatory responses which, in turn, could play a pivotal role in promoting a pro-regenerative environment. On another front, proteins identified in clusters related to hemostasis and extracellular matrix organization hold critical importance in processes like endothelial cell recruitment and matrix remodeling during wound healing. These processes influence the matrix-cell interactions and recruit cell progenitors involved in re-epithelialization and angiogenesis. This holistic view, particularly focused on the protein phenotypes, offers valuable insights for understanding the functional impact of the hAMSCs secretome in regenerative medicine. It paves the way for potential breakthroughs in harnessing the therapeutic potential of hAMSC.

### Supplementary Information

**Additional file 1**: **Table S1.** Protein identification data for hAMSCs secretome pools 1-4

**Additional file 2**: **Table S2.** Proteins identification data for DMEMF12 samples (CTRL 1-3) analyzed against Homo sapiens and Bos Taurus databanks (A-F), proteins common to all samples (G) and summary of relative albumin fragments identification data (H)

**Additional file 3**: **Table S3.** Out of the 157 proteins common to secretome pools 1-4, these proteins have been classified as secreted proteins based on the grouping analysis against the Human Protein Atlas secretome database (B) and Sub-categories distribution of the proteins classified as secreted (C)

**Additional file 4**: **Table S4.** Classification of the 157 common proteins was carried out using the Human Protein Atlas placenta proteome database as a reference. The relative distribution is based on tissue expression. Furthermore, the grouping analysis shows that the proteins within this group are also classified as secreted proteins

**Additional file 5**: **Table S5.** Protein average area values of the 157 proteins common to the hAMSCs secretome pools 1-4 (A), and the list of the 33 most abundant proteins in the hAMSCs secretome (average area in the range1xE9-5xE7 in decreasing order) (B).

## Data Availability

The mass spectrometry proteomics data have been deposited to the ProteomeXchange Consortium via the PRIDE (http://www.ebi.ac.uk/pride) [90] partner repository with the dataset identifier PXD041088.

## Abbreviations

FASP: Filter-aided sample preparation
LC–MS: Liquid chromatography-high resolution mass spectrometry
hAMSCs: Human amniotic membrane mesenchymal stromal cells
ECM: Extracellular matrix
ELISA: Enzyme-linked immunosorbent assays
FBS: Fetal bovine serum
IAA: Iodoacetamide
DTT: D,L-dithiothreitol (DTT)
AMBIC: Ammonium bicarbonate
FA: Formic acid
UHPLC-ESI–MS/MS: Ultra-high-performance liquid chromatography-electrospray mass spectrometry
CID: Collision-induced dissociation
DDS: Data-dependent scan
CTRL: Control of reference
FDR: False discovery rate
HPP: Human proteome project
GO: Gene ontology
CM: Conditioned medium
hAMSC-CM: Human amniotic membrane mesenchymal stromal cells conditioned medium
HPA: Human protein atlas
MSC: Mesenchymal stromal cells
